# Novel multimorbidity clusters in people with eczema and asthma: a population-based cluster analysis

**DOI:** 10.1038/s41598-022-26357-x

**Published:** 2022-12-18

**Authors:** Amy R. Mulick, Alasdair D. Henderson, David Prieto-Merino, Kathryn E. Mansfield, Julian Matthewman, Jennifer K. Quint, Ronan A. Lyons, Aziz Sheikh, David A. McAllister, Dorothea Nitsch, Sinéad M. Langan

**Affiliations:** 1 Department of Non-Communicable Disease Epidemiology, London School of Hygiene and Tropical Medicine, Keppel Street, London, WC1E 7HT UK; 2 National Heart and Lung Institute, Imperial College London, London, UK; 3 National Centre for Population Health and Wellbeing Research, Swansea University Medical School, Swansea, UK; 4 Administrative Data Research UK, Swansea University Medical School, Swansea, UK; 5 Asthma UK Centre for Applied Research, Usher Institute, University of Edinburgh, Teviot Place, Edinburgh, EH8 9DX UK; 6 Institute of Health and Wellbeing, University of Glasgow, Glasgow, UK; 7 Health Data Research UK, Gibbs Building, 215 Euston Road, London, NW1 2BE UK

**Keywords:** Epidemiology, Asthma, Atopic dermatitis

## Abstract

Eczema and asthma are allergic diseases and two of the commonest chronic conditions in high-income countries. Their co-existence with other allergic conditions is common, but little research exists on wider multimorbidity with these conditions. We set out to identify and compare clusters of multimorbidity in people with eczema or asthma and people without. Using routinely-collected primary care data from the U.K. Clinical Research Practice Datalink GOLD, we identified adults ever having eczema (or asthma), and comparison groups never having eczema (or asthma). We derived clusters of multimorbidity from hierarchical cluster analysis of Jaccard distances between pairs of diagnostic categories estimated from mixed-effects logistic regressions. We analysed 434,422 individuals with eczema (58% female, median age 47 years) and 1,333,281 individuals without (55% female, 47 years), and 517,712 individuals with asthma (53% female, 44 years) and 1,601,210 individuals without (53% female, 45 years). Age at first morbidity, sex and having eczema/asthma affected the scope of multimorbidity, with women, older age and eczema/asthma being associated with larger morbidity clusters. Injuries, digestive, nervous system and mental health disorders were more commonly seen in eczema and asthma than control clusters. People with eczema and asthma of all ages and both sexes may experience greater multimorbidity than people without eczema and asthma, including conditions not previously recognised as contributing to their disease burden. This work highlights areas where there is a critical need for research addressing the burden and drivers of multimorbidity in order to inform strategies to reduce poor health outcomes.

## Introduction

Asthma and eczema (also known as atopic eczema or atopic dermatitis^1^) are common allergic diseases: in 2019 they affected 4% (asthma) and 10% (eczema) of the adult U.K. population^2^ and were globally the 24th^3^ and 28th^4^ largest causes of years lived with a disability. Asthma and eczema have major adverse impacts on quality of life and place considerable financial burdens on health services and individuals^5–9^.

Substantial, well-documented comorbidity exists amongst allergic diseases^10–18^: asthma, eczema, hay fever and food allergies, in particular, often cluster in some combination in the same individuals. This is allergic multimorbidity, a subset of multimorbidity in a more general sense, which occurs when an individual accumulates two or more chronic conditions^19^. The pace of multimorbidity research has accelerated in recent years, and many recent studies have shed light on clustering patterns of chronic conditions, especially in general or older-age populations^20–22^.

Evidence is now accumulating for increased risks of non-allergic conditions in people with allergic conditions, such as cardiovascular disease and fractures^23–27^. Thus, individuals with allergic conditions may be at greater risk for multimorbidity, beyond allergic multimorbidity, than previously understood. Given evidence for associations with conditions not traditionally considered chronic^25,28,29^, they may be at greater risk for morbidity patterns that go beyond even traditional multimorbidity.

If these patterns differ from those in people without allergic conditions, this may suggest a need to employ additional or alternative disease prevention and management strategies. To date, multimorbidity has not been studied in people with existing asthma or eczema, although it has been studied more broadly in people with chronic obstructive airway disease^30^ and in people with other non-allergic conditions such as suspected acute coronary syndrome^31^ and type 2 diabetes^22^. Thus, there is a need to better understand what other conditions tend to cluster together in people with allergic conditions.

We set out to identify clusters of multimorbidity, including both chronic and non-chronic conditions, in U.K. adults with asthma and (separately) with eczema using a hypothesis-free study design. Like some multimorbidity researchers, we look for clusters of diseases^21,22,32,33^, rather than clusters of individuals that are retrospectively assessed for disease characteristics^20,21,30,31^. However, there are some differences with our method. First, previous research undertaken in populations with an index disease ignored possible multimorbidity differences with the non-diseased population, identifying morbidity clusters in only people with the specific exposures^22,30,31^. Our approach compares morbidity clusters in asthma and eczema to clusters identified in populations without these conditions and is hypothesis generating, highlighting key areas where research focus is warranted. Second, many others looking for disease clusters have identified them using metrics such as tetrachoric correlations^21,32^ or relative measures of association between conditions^22^, which, while useful in providing quantitative estimates, suffer from limited clinical interpretability. We cluster on probabilities of comorbid disease (similar to one analysis by Roso-Llorach et al.^32^) so that the clusters have clinically understandable interpretations.

## Methods

### Data source

We used routinely-collected electronic health records from U.K. primary care. The Clinical Practice Research Datalink (CPRD) GOLD includes de-identified health record data from participating general practices covering over 11.3 million patients from 674 practices in the U.K., and is broadly representative of the U.K. population with respect to age, sex, ethnicity, and geographic region^34^. See Online Supplement Note S1 for full inclusion criteria.

### Exposures

We identified people with eczema (who may also have asthma) based on an existing algorithm previously validated in CPRD^35^ requiring at least one diagnostic code for eczema and at least two records for eczema therapies recorded on separate days. Similarly, we identified people with asthma (who may also have eczema) based on an existing validated algorithm^36^ of ever having an asthma morbidity code recorded in primary care. Codelists for identifying these exposures and the following outcomes are publicly available^37^. Although eczema and asthma are relapsing and remitting diseases, individuals were included in this study if they ever met these definitions during the active CPRD observation window, regardless of recency. We matched each individual with eczema or asthma to up to five controls, with replacement, on age (within 5 years), sex and general practice. Full details of study participant identification are available in Supplementary Fig. S2.

### Outcomes

Our outcomes were first records from broad diagnostic categories recorded at any time in an individual’s medical history up to July 2020. Historical records reached back to 1915 and 1916 in the eczema and asthma cohorts, respectively. U.K general practices contributing to CPRD GOLD record patient contacts using Read morbidity codes, whose chapters are similar to those of the International Classification of Diseases (ICD) and are organized at the highest level alphanumerically. We used chapters A to U, which are categorized by body systems (Table 1), and excluded the numeric chapters containing history and examination findings. Not all chapters represent chronic conditions (e.g. A, Infectious/parasitic diseases), but we include them so we can see a high-level overview of the burden of disease in people with eczema and asthma.

**Table 1:** Characteristics of the study populations.

	Eczema	Matched without Eczema	Asthma	Matched without Asthma
Total	434,422	1,333,281	517,712	1,601,210
Participants in both cohorts (asthma and eczema)	115,685 (26.6)	696,623 (52.2)	115,685 (22.3)	696,623 (43.5)
Participants in asthma or eczema cohort only	318,737 (73.4)	636,658 (47.8)	402,027 (77.7)	904,587 (56.5)
Median age (SD)	47 (20.6)	47 (19.6)	44 (19)	45 (18.8)
18–20	15,157 (3.5)	36,035 (2.7)	13,321 (2.6)	37,912 (2.4)
21–40	145,884 (34)	429,789 (32)	197,787 (38)	550,756 (34)
41–60	125,195 (29)	426,218 (32)	162,946 (31)	540,549 (34)
61–80	108,912 (25)	337,987 (25)	112,237 (22)	370,734 (23)
80 +	39,038 (9)	102,638 (7.7)	31,276 (6)	100,829 (6.3)
NA	236 (0.054)	614 (0.046)	145 (0.028)	430 (0.027)
**Sex**				
Female	251,251 (58)	738,279 (55)	276,943 (53)	844,582 (53)
Male	183,171 (42)	595,002 (45)	240,769 (47)	756,628 (47)
**Primary care records by read chapter**				
A: Infectious/parasitic diseases	312,098 (72)	670,680 (50)	314,199 (61)	783,263 (49)
B: Neoplasms	149,493 (34)	369,973 (28)	150,796 (29)	421,313 (26)
C: Endocrine/metabolic	156,719 (36)	388,373 (29)	167,911 (32)	433,407 (27)
D: Blood diseases	59,748 (14)	126,129 (9.5)	57,362 (11)	138,301 (8.6)
E: Mental disorders	208,728 (48)	504,918 (38)	243,290 (47)	581,697 (36)
F: Nervous system/senses	342,550 (79)	811,457 (61)	359,420 (69)	933,042 (58)
G: Circulatory system	197,157 (45)	503,260 (38)	204,660 (40)	570,626 (36)
H: Respiratory system	371,600 (86)	888,421 (67)	510,971 (99)	972,222 (61)
J: Digestive system	267,700 (62)	624,300 (47)	289,606 (56)	715,687 (45)
K: Genito-urinary system	277,638 (64)	672,914 (50)	288,996 (56)	765,885 (48)
L: Pregnancy/childbirth/puerperium	91,275 (21)	246,357 (18)	102,298 (20)	290,132 (18)
M: Skin/subcutaneous	434,422 (100)	840,711 (63)	392,631 (76)	1,022,333 (64)
N: Musculoskeletal	339,610 (78)	875,221 (66)	372,890 (72)	1,009,574 (63)
P: Congenital anomalies	30,115 (6.9)	67,266 (5)	34,130 (6.6)	81,081 (5.1)
Q: Perinatal conditions	13,185 (3)	22,396 (1.7)	13,034 (2.5)	26,547 (1.7)
R: Ill-defined conditions/working diagnoses	305,969 (70)	701,401 (53)	320,170 (62)	788,894 (49)
S: Injury/poisoning	303,537 (70)	745,678 (56)	346,350 (67)	877,169 (55)
T: Causes of injury/poisoning	138,165 (32)	300,147 (23)	149,267 (29)	339,009 (21)
U: External causes of morbidity and mortality	30,276 (7)	69,687 (5.2)	40,235 (7.8)	81,575 (5.1)

All numbers are n (%) unless otherwise specified.

### Statistical analysis

We compared patterns of Read chapter clusters (multimorbidity) in groups of people with and without eczema, and with and without asthma. Our intention was to generate future research hypotheses, so our method of comparison was informal: we identified morbidity clusters both in exposed individuals and in controls, and visually inspected differences in absolute probabilities of multimorbidity between them. We did not apply statistical tests as we did not wish to make final judgements on these clusters based on exploratory work.

We sought to identify clusters using a disease-driven approach, clustering on summary ‘disease-level’ data derived from individual data; i.e., we directly identified diseases tending to cluster together rather than first identifying individuals clustering together and retrospectively assessing which diseases they had. A feature of this method is that the resulting clusters are not discrete groups of people, but are instead networks of relationships between diseases, which are determined by clinically relevant cutoffs. Lower cutoffs (using the metric we describe below) make larger clusters because they include weaker relationships, and higher cutoffs make smaller clusters because they restrict to the strongest relationships. An advantage of this method is that each disease can be considered in the context of all the others, even if the association is small, and rare diseases can be given more weight (in some circumstances, see below). This is useful for hypothesis-generation and for reducing the risk of overlooking clusters containing rare diseases, which can happen when clustering individuals.

We derived clusters using a two-step approach. In the first step, we used individual-level data to estimate the Jaccard index^38–40^ between each pair of Read chapters, i.e., the probability of an individual having a record in both chapters given having a record in one. We did this with mixed-effects logistic regression, excluding individuals from each regression who had records in neither chapter of the pair. In this way, the population denominators reduce and it is easier to see associations between diseases that could be overlooked when the populations contain large numbers of people who have neither disease. Explanatory variables were time from the first chapter record to the end of follow-up, age at the first chapter record, sex, eczema or asthma exposure (as relevant), and a random practice intercept.

From the parameters estimated in these models, we predicted the mean Jaccard index in adult populations with five years’ of followup from first chapter morbidity recording at an average practice (i.e. zero additional practice effect). We chose populations at two ages of first chapter recording (18 and 50 years; selected for clinical interest) for both sexes in each exposure group. We split our analyses by age and sex because we believe these demographics modify multimorbidity. Older people have had a longer time to accumulate morbid conditions than younger people, and we expect them to have larger clusters. Similarly, under the Read system, only women can have a diagnosis in Chapter L (pregnancy/childbirth/puerperium) while other chapters, like many diseases, could reasonably be expected to be more common in one sex or the other. This could affect the closeness (Jaccard index) between them and thus the cluster size or composition.

We performed network and hierarchical cluster analyses at each combination of age, sex and eczema or asthma exposure status using the Jaccard distance (1 minus Jaccard index). To produce the undirected network graphs we used the igraph R package^41^ and restricted the display of edges (the degree of correlation between a pair of Read chapters) to those with Jaccard distance > 30%. The 30% probability was selected to represent a clinically meaningful risk worth monitoring in individuals with existing morbidity. We performed hierarchical cluster analysis using the complete-linkage agglomeration method, where each Read chapter begins in its own cluster (Jaccard distance equal to 1; the record in one chapter has a 100% probability of co-occurring with other chapters in the cluster) and as the Jaccard distance decreases, the chapters combine into larger clusters of decreasingly closely-related chapters until all form a single cluster (Jaccard distance equal to 0; all chapters have a 0% or higher probability of co-occurring with other chapters in the cluster). We visualised the results with a dendrogram highlighting a cutoff at 30%, representing the conditional probability of having a morbidity code in one chapter from the cluster within 5 years, given the occurrence of a morbidity code in a different chapter from the cluster.

To check sensitivity of our findings, we repeated our hierarchical cluster analyses after rerunning our regression models with one and 10 years of followup, and by using the Ward linkage algorithm.

Data management was performed in STATA version 15^42^ and statistical analyses in R version 4.0.5^43^. This article follows RECORD^44^ reporting guidelines.

### Ethical approval

This work did not involve experimentation in humans or animals or the use of human tissue samples. It is an analysis of de-identified electronic health records provided by the Clinical Practice Research Datalink (CPRD) in the UK, which has practice-level consent to use pseudonymised patient data for research. The current standard practice for the use of pseudonymised data is adopted by CPRD and does not require consent. However, CPRD works with contributing practices to ensure patients are aware of such use of their data and of their right to dissent from the use of their pseudonymised data if they wish (05/MRE04/87). All methods were performed in accordance with the relevant guidelines and regulations set out by CPRD. The CPRD Independent Scientific Advisory Committee (20_000259A) and London School of Hygiene and Tropical Medicine Research Ethics Committee (26602) approved this work.

### Role of the funding source

This work was funded by grants from the UK Medical Research Council (MR/V005146/1), the Innovative Medicines Initiative 2 Joint Undertaking (JU) (821511 (BIOMAP)) and the Wellcome Trust (205039/Z/16/Z) and was supported by Health Data Research UK. The funders had no role in study design; data collection, analysis, or interpretation; report writing; or the decision to submit the paper for publication.

## Results

### Study population

We included 434,422 individuals with eczema (58% female, median age 47 years) and 1,333,281 matched controls without eczema (55% female, 47 years), and 517,712 individuals with asthma (53% female, 44 years) and 1,601,210 matched controls without asthma (53% female, 45 years) (Table 1, Supplementary Fig. S2). Many controls (n = 696,623) were matched both to individuals with eczema (52% of total) and to individuals with asthma (57% of total) and contributed to both analyses. Similarly, many people were identified as having both eczema and asthma (n = 115,685, representing 27% of people with eczema, and 22% of people with asthma) and contributed to both analyses.

### Prevalence of morbidities

**Figure 1:**
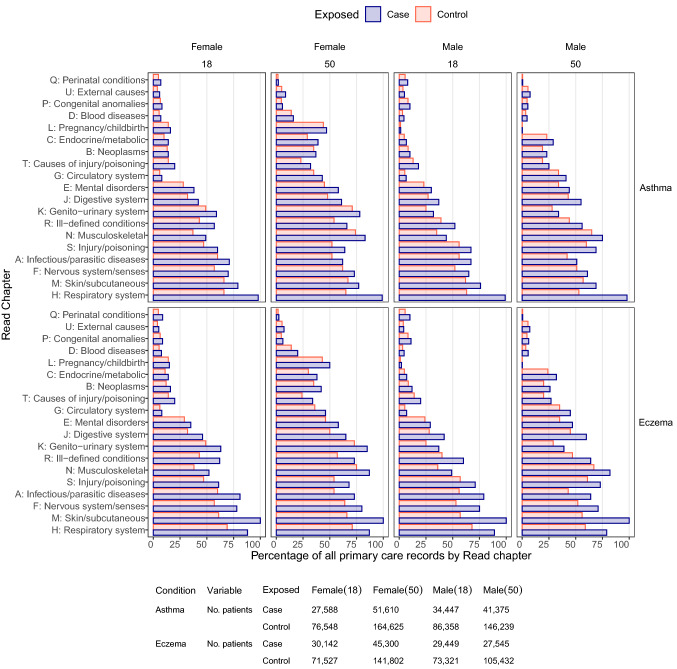
Proportion of people with asthma (top 4 graphs) and eczema (bottom 4 graphs) and their matched controls that ever recorded a diagnostic code in each Read chapter by age (18 ± 5 years, 50 ± 5 years) and sex.

Excluding skin diseases (Read Chapter M), the most common morbidity codes ever recorded in individuals with eczema were for Respiratory and Nervous system conditions (chapters H and F, 86% and 79%). For matched controls without eczema, including all chapters, they were for Respiratory and Musculoskeletal conditions (H and N, 67% and 66%) (Table 1, Supplementary Fig. S3), although this varied by age and sex (Fig. 1).

Excluding respiratory diseases (Chapter H), the most common ever-recorded codes for individuals with asthma were for Skin and Musculoskeletal conditions (Chapters M and N, 76% and 72%). Matched controls without asthma were the same but less prevalent (64% and 63%). All chapters were more commonly recorded in people with eczema and asthma compared with their controls.

### Comparison of multimorbidity

Summary statistics from 171 regression models (one for each pairwise combination of Read chapters) for each condition are available in Supplementary Figs. S4–S5. People with eczema or asthma generally had their first recorded diagnosis from either Read chapter earlier than people without, more often experienced the regression outcomes of having both diagnoses, and were followed for a longer time (Supplementary Figs. S4–S5). Sex was well-balanced between populations with and without eczema and asthma.

We found larger multimorbidity clusters in people with eczema and people with asthma compared with controls. Detailed images of networks and dendrograms are available in Supplementary Figs. S6–S13.

### Undirected networks

**Figure 2:**
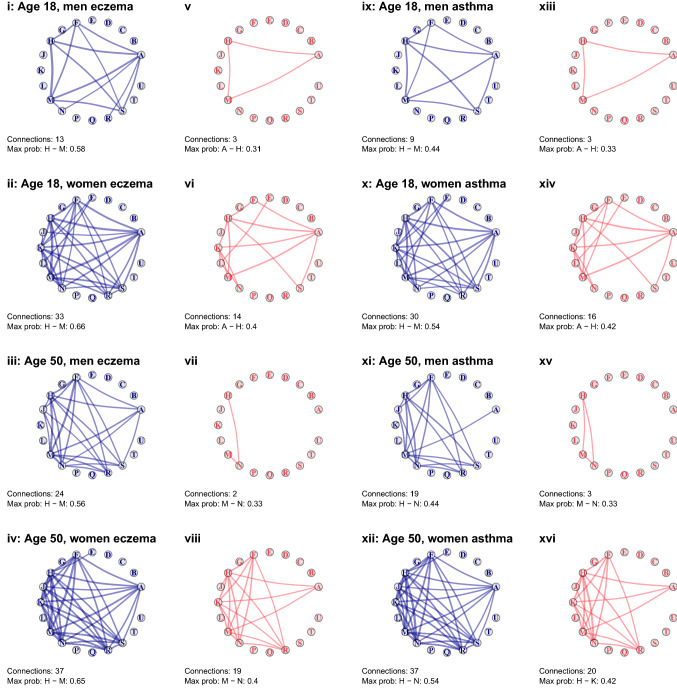
Undirected networks of Read code chapter diagnoses in people with eczema (A–D), without eczema (E–H), with asthma (J–M) and without asthma (N–Q). Edges displayed are those that represent ≥ 30% probability of Read code chapter recording co-occurrence within 5 years. A: Infectious/parasitic diseases, B: Neoplasms, C: Endocrine/metabolic, D: Blood diseases, E: Mental disorders, F: Nervous system/senses, G: Circulatory system, H: Respiratory system, J: Digestive system, K: Genito-urinary system, L: Pregnancy/childbirth, M: Skin/subcutaneous, N: Musculoskeletal, P: Congenital anomalies, Q: Perinatal conditions, R: Ill-defined conditions, S: Injury/poisoning, T: Causes of injury/poisoning, U: External causes.

Figure 2 summarises 16 undirected networks of Read chapters by (modelled) age at first recorded condition, sex and exposure/control status for eczema and asthma groups. The Read chapters are fixed in the same place for all groups so that the morbidity associations can be visually compared easily between groups. There was greater interconnectedness in populations with eczema and asthma compared with controls, in women compared to men, and in older diagnostic ages compared to younger.

The most connectivity between Read chapters was in women with eczema or asthma (between 30 and 37 edges, Panels ii, iv, x, xii), followed by men with eczema or asthma (9–24 edges, Panels i, iii, ix, xi), female controls (14–20, Panels vi, viii, xiv, xvi) and male controls (2 or 3, Panels v, vii, xiii, xv). In general, people aged 50 at first recorded condition had greater five-year multimorbidity than people aged 18 of the same sex.

### Hierarchical cluster analyses

**Figure 3:**
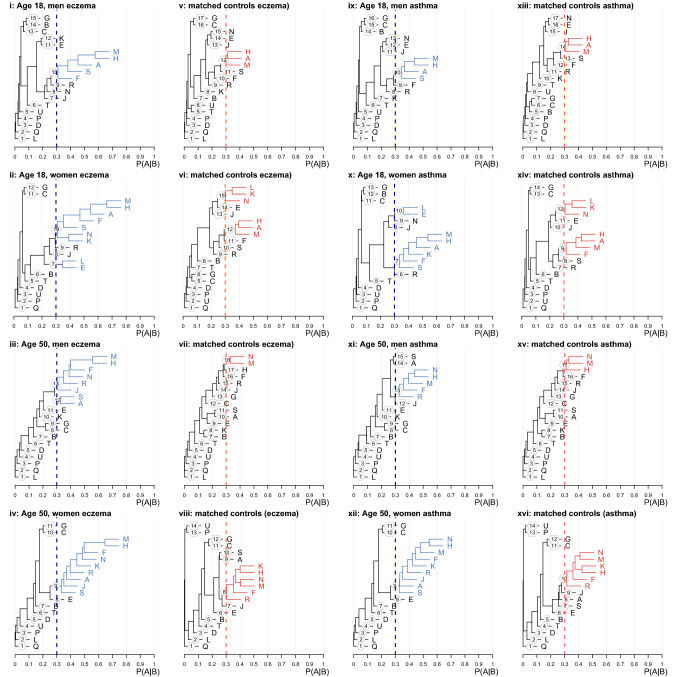
Dendrograms showing Read code chapters that cluster together in men and women with eczema (panels A–D) and without eczema (E–H) and in men and women with asthma (J–M) and without asthma (N–Q). Clusters of chapters with a greater than 30% probability of co-occurrence within 5 years are shown in color (Blue, eczema/asthma. Red, controls). All probabilities are calculated at 5 years’ followup from the first chapter recording. A: Infectious/parasitic diseases, B: Neoplasms, C: Endocrine/metabolic, D: Blood diseases, E: Mental disorders, F: Nervous system/senses, G: Circulatory system, H: Respiratory system, J: Digestive system, K: Genito-urinary system, L: Pregnancy/childbirth, M: Skin/subcutaneous, N: Musculoskeletal, P: Congenital anomalies, Q: Perinatal conditions, R: Ill-defined conditions, S: Injury/poisoning, T: Causes of injury/poisoning, U: External causes.

Table 2 and Fig. 3 present results from the hierarchical cluster analyses. In Fig. 3, the closely related Read chapters are grouped together so that the clusters can be easily visualised.

**Table 2:** Clusters of multimorbidity in people with eczema or people with asthma and controls without eczema or without asthma.

	Eczema/asthma	Controls
Men, 18 years at first morbidity diagnosis	Cluster 1 M: Skin/subcutaneous tissue H: Respiratory system A: Infections/parasitic diseases S: Injury/poisoning F: Nervous system/senses (eczema only)	Cluster 1 M: Skin/subcutaneous tissue H: Respiratory system A: Infections/parasitic diseases
Men, 50 years at first morbidity diagnosis	Cluster 1 M: Skin/subcutaneous tissue H: Respiratory system N: Musculoskeletal F: Nervous system/senses R: Ill-defined conditions J: Digestive system (eczema only) Cluster 2. (cluster in eczema only) A: Infections/parasitic diseases S: Injury/poisoning	Cluster 1 M: Skin/subcutaneous tissue H: Respiratory system (asthma controls only) N: Musculoskeletal
Women, 18 years at first morbidity diagnosis	Cluster 1 M: Skin/subcutaneous tissue H: Respiratory system A: Infections/parasitic diseases F: Nervous system/senses S: Injury/poisoning K: Genito-urinary system N: Musculoskeletal (eczema only) Cluster 2 L: Pregnancy/childbirth/puerperium E: Mental disorders	Cluster 1 M: Skin/subcutaneous tissue H: Respiratory system A: Infections/parasitic diseases F: Nervous system/senses (asthma controls only) Cluster 2 L: Pregnancy/childbirth/puerperium N: Musculoskeletal K: Genito-urinary system
Women, 50 years at first morbidity diagnosis	Cluster 1 M: Skin/subcutaneous tissue H: Respiratory system F: Nervous system/senses N: Musculoskeletal K: Genito-urinary system R: Ill-defined conditions A: Infections/parasitic diseases S: Injury/poisoning J: Digestive system	Cluster 1 M: Skin/subcutaneous tissue H: Respiratory system F: Nervous system/senses N: Musculoskeletal K: Genito-urinary system R: Ill-defined conditions

In men with eczema, modelled with first condition occurring at age 18, we identified one cluster of five-year multimorbidity comprising Skin, Respiratory, Infectious/parasitic and Neurological conditions and Injuries/Poisoning (Read chapters M/H/A/F/S; Panel i). In their controls without eczema, we identified a smaller cluster containing the chapters for Skin, Respiratory and Infectious/parasitic conditions only (M/H/A; Panel v). When first condition was modeled at age 50, we identified two clusters in men with eczema (Panel iii) containing all the conditions from the earlier diagnostic age—Infectious/parasitic diseases and Injury/Poisoning (A/S; smaller cluster) and Skin, Respiratory and Neurological conditions (M/H/F; larger cluster)—as well as Musculoskeletal, Ill-defined, and Digestive system conditions (N/R/J) in the larger cluster. In their controls, we found a single, small cluster of Skin and Musculoskeletal conditions (M/N) (Panel vii).

In women with eczema, modeled with the first condition occurring at age 18, we identified two clusters of five-year multimorbidity (Panel ii). The larger cluster contained many of the same five conditions as the clusters from men with eczema (Skin, Respiratory, Infectious/Parasitic, Neurological and Injury/Poisoning; chapters M/H/A/F/S), plus Musculoskeletal and Genito-urinary conditions (N/K). The smaller contained Mental Health and Pregnancy/Childbirth/Puerperium conditions (E/L). In their controls we also identified two clusters but with different patterns (Panel vi). One was a subset of the larger eczema cluster, containing Skin, Respiratory and Infectious/parasitic conditions (M/H/A); the other contained Pregnancy/Childbirth/Puerperium, Genito-urinary and Musculoskeletal conditions (L/K/N). When first condition was modeled at age 50, we identified a single large cluster of nine Read chapters; Skin, Respiratory, Infectious/parasitic, Injury/Poisoning, Neurological, Musculoskeletal and Genito-urinary (M/H/A/S/F/N/K, i.e. the same conditions occurring in the younger diagnostic age), as well as Ill-defined conditions and Digestive system disorders (R/J) (Panel iv). In controls we found a larger multimorbidity cluster than in men or female controls with first diagnosis at age 18, but smaller than their matches, including Skin, Respiratory, Neurological, Musculoskeletal, Genito-urinary and Ill-defined conditions (M/H/F/N/K/R) (Panel viii).

Clusters identified in people with asthma were very similar to those identified in people with eczema (Panels ix-xii), and clusters identified in asthma controls were very similar to those identified in eczema controls (Panels xiii-xvi). Table 2 details the differences, all of which are due to chapters or clusters of chapters falling just below 30% co-occurrence probability in eczema and just above it in asthma or vice versa.

### Sensitivity analyses

For both eczema and asthma populations, and their controls, increasing the length of follow-up generally increased the probability of each pair of co-occurrences, producing larger and/or more clusters at the 30% cutoff (Supplementary Figs. S14A–H and S15A–H). Switching to Ward linkage algorithms in the hierarchical clustering method had no material impact on the clusters identified (Suppl. Fig. S16).

## Discussion

In this hypothesis-free exploratory study of morbidity clustering in U.K. adults with eczema and adults with asthma, we found clusters of five-year multimorbidity that were larger than those observed in controls. Cluster size and composition were related to age at diagnosis of first condition, sex and length of follow-up. Populations with eczema and asthma experienced greater multimorbidity than controls, and amongst them women had more co-existing diagnoses than men, people who were older at the age of their first diagnosed condition had more than those who were younger, and many clusters grew larger with more follow-up time. Read Chapters S (Injury/poisoning), J (Digestive system), and E (Mental disorders) were observed in some eczema and asthma clusters, but none of the control clusters. Chapter F (Nervous system/senses) was observed more often in clusters for people with eczema or asthma than controls, especially in men.

Allergic conditions are common in the U.K. population, and eczema and asthma carry a large disease burden. This study suggests a potential increased health burden of non-allergic diseases in populations with eczema or asthma compared to those without, beyond those that are currently recognised.

### Strengths and limitations

To our knowledge, this is the first exploration of clusters of multimorbidity in eczema and asthma populations using UK routine health data. We used data up to July 2020, so our findings reflect contemporary health needs in adults with and without eczema and asthma. In the UK healthcare setting, the GP is gatekeeper for all types of care and there is good evidence that major outcomes recorded outside of primary care make their way into GP records^34,45,46^.

Our study was large, including approximately half a million people each with eczema and asthma (with about a quarter of each group included in the other) and over 50 million recorded diagnoses in each group. Our source population (CPRD) is representative of the UK population in terms of age, sex, and socio-economic deprivation levels^34^, so our findings can be generalised to people with these conditions in the U.K. We stratified clusters on age and sex, so we were able to show how multimorbidity could vary by age and sex. Further, by basing clusters on the absolute probabilities of comorbidities within exposed individuals and controls, we were able to provide a more detailed assessment of absolute risks within each group (and consequently offer a means of assessing how clinically meaningful differences between them might be). Our method of clustering on diseases rather than individuals gave potential to see associations with rare diseases (in some circumstances; see limitations below) that might otherwise be missed. We identified people with eczema and asthma based on validated algorithms^35,36^ and we have made all our code lists and analysis code available^37^.

However, our study had limitations. There is the possibility of misclassification bias, particularly if asthma, eczema and comorbidities were not available, not recorded, misrecorded or recorded at the wrong time in electronic health records. We may have classified some people with likely milder eczema or asthma as controls, which may have confounded our findings if multimorbidity is different in these two populations. Similarly, if multimorbidity varies by eczema endotype or subtype, our analysis would not have picked this up and our multimorbidity clusters may be confounded by subtype. We did not consider the temporal direction of associations between morbidities so we cannot draw causal inferences on the clusters we identified, and further research is required to disentangle iatrogenic morbidities (e.g., steroid induced osteoporosis predisposing to fractures) from other more aetiologically informative associations. However, by removing temporal constraints we were not limited by potential inaccuracies in the timing of diagnoses, and were able to consider the totality of participants’ primary care records including iatrogenic effects. A critical point is that our goal was not to assess solely morbidity related to asthma or eczema themselves, but to additionally capture morbidities that may have resulted from treatment.

Although we consider sex and age as potential modifiers of morbidity clustering, additional aspects such as educational attainment and employment status may also be important^47^, and could not be captured in this data source. Socioeconomic status (SES), proxied by GP practice in our data, was included in our statistical model but, because it suffers from limited clinical interpretability, we were unable to stratify by it and our clusters represent an average SES. Our use of broad diagnostic categories limited our ability to make recommendations for clinical practice, which would have required greater diagnostic granularity. We accounted for one recorded event per person per Read chapter, masking any additional burden or variety of disease some people may have had with multiple events recorded under one chapter. Our data included the first three months of the Covid-19 pandemic, where primary care consultations reduced dramatically and some incident conditions may not have been recorded; however, three months is at maximum 1.4% of the life course of individuals in this study (of an 18-year-old) so this is unlikely to have had substantial effects on our findings.

Although we matched controls individually to people with eczema or asthma, we broke matching in our regression analyses because we excluded individuals with no relevant recorded events. Consequently, we modelled matching factors directly and reported age- and sex-stratified multimorbidity clusters rather than population clusters. We matched with replacement, so there could be some dependence in observations where individuals were sampled more than once, although this is probably mitigated somewhat by these exclusions.

Our statistical models contained few covariates and we did not consider covariate interactions or nonlinear effects. We used the Jaccard distance to measure the closeness of two morbidities, but it has limitations when comparing very common diseases with rare diseases (Supplementary Fig. S17). Thus the (arbitrary) choice of a 30% threshold for associations across all pairs of chapters, which may be reasonable for comparing diseases with similar prevalences, could be too conservative when comparing diseases with different prevalences and we may have missed some important associations.

### Results in context

Previous work on multimorbidity in eczema and asthma has focused on the co-occurrence of asthma, eczema, rhinitis, and food allergies^48^. The co-occurrence of allergic conditions is supported by shared biological pathways^12^. Many studies have highlighted the high burden of polypharmacy and associated costs in people with asthma and allergic conditions^11^. A study using National Health and Nutrition Examination Survey (NHANES) data documented that half of the US population diagnosed with asthma had co-occurring conditions (including hypertension and arthritis) and that these comorbid conditions were associated with increased emergency room attendance^50^. Our finding of clusters of conditions in people with eczema and asthma not seen as strongly in controls requires further exploration to understand specific associations and their mechanisms, and supports multimorbidity being an important consideration in allergic disease.

Key insights from this study include associations with injury, mental health conditions and disorders of the digestive system. Previous research has identified increased risks of bone-related outcomes, including fractures and injury in people with eczema and asthma, which may be partly mediated through treatments, including use of oral corticosteroids and sedating antihistamines^25,28,29,51,52^. Existing research has also identified an association between severe asthma and underlying mental health conditions^53^, even suggesting that an asthma diagnosis may be incorrect for some people and their symptoms (dysfunctional breathing) caused instead by a psychiatric morbidity (e.g. anxiety). Associations between atopic disorders and digestive disorders such as eosinophilic oesophagitis are well described, but probably underdiagnosed, and require further population-based evidence^54^. Older literature considers the hypothesis that acute stress can worsen eczema and that some specific asthma phenotypes have a neurological basis^55^, but to date there is limited epidemiological research to support these theories. Although associations with individual health conditions are known and have face validity, the key insights from this research are about the coexistence of conditions involving different body systems, requiring different evaluations and therapies for holistic care.

### Implications for clinical practice, policy and further research

Our work gives a new understanding of multimorbidity in populations with eczema and asthma and suggests the need for holistic approaches in care and research. However, it is only a first look into this question. More research is needed to identify which specific diagnoses are contributing to the higher-level recordings in the novel clusters, with a specific focus on novel components, including neurological and digestive disorders, and what underlying mechanisms may explain them before clinical care recommendations can be made. In order to understand mechanisms, insight into the temporality of developing morbidities (trajectories) and associations of multimorbidity clusters with disease severity and treatment would be critical, followed by triangulation with other data types including genetic data to help understand causal mechanisms.

This study therefore highlights a need for further research of non-allergic diseases in populations with eczema and asthma to improve health and reduce poor outcomes.

## Supplementary Information

Supplementary Information.

## Data Availability

The data that support the findings of this study were obtained under a single-use licence by the UK CPRD and cannot be shared by the authors. However, interested researchers can request the data under a new licence by following the procedures outlined at https://cprd.com/data-access. All data management and analysis computer code is available via GitHub (https://github.com/hendersonad/2020_multimorbidity) and archived in Zenodo (https://doi.org/10.5281/zenodo.7307504). All code is shared without investigator support.
