# Supplementary material for: Novel multimorbidity clusters in people with eczema and asthma: a population-based cluster analysis
Source: Sci Rep. 2022 Dec 18;12:21866. doi: 10.1038/s41598-022-26357-x (PMC9760185; doi:10.1038/s41598-022-26357-x)
Supplement: Supplementary file 1 — Supplementary Information. [file 41598_2022_26357_MOESM1_ESM.pdf]

Online appendix for: Novel multimorbidity clusters in people with eczema and asthma: a population-based cluster analysis

Authors: Amy R Mulick[1] MSc,* Alasdair D Henderson[1] PhD,* David Prieto-Merino[1] PhD,* Kathryn E Mansfield[1] PhD, Julian Matthewman[1] MSc, Jennifer K Quint[2] PhD, Ronan A Lyons[3,4] PhD, Aziz Sheikh[5] MD, David A McAllister[6] PhD, Dorothea Nitsch[1] PhD, Sinéad M Langan[1,7] PhD

* Contributed equally as joint first authors

Affiliations:
[1] Department of Non-Communicable Disease Epidemiology, London School of Hygiene and Tropical Medicine, Keppel Street, London, WC1E 7HT, United Kingdom
[2] National Heart and Lung Institute, Imperial College London, London, United Kingdom
[3] National Centre for Population Health and Wellbeing Research, Swansea University Medical School, Swansea, United Kingdom
[4] Administrative Data Research UK, Swansea University Medical School, Swansea, United Kingdom
[5] Asthma UK Centre for Applied Research, Usher Institute, University of Edinburgh, Teviot Place, Edinburgh, EH8 9DX, UK
[6] Institute of Health and Wellbeing, University of Glasgow, Glasgow, UK
[7] Health Data Research UK, Gibbs Building, 215 Euston Road, London, NW1 2BE, United Kingdom

Supplementary material: 1 note, 17 figures

SUPPLEMENTARY MATERIAL

Supplementary Note 1. Study population

Individuals in CPRD-GOLD were eligible for inclusion in this study if, by the end of the control matching period (see below), they: were at least 18 years old, had at least one year registration with an eligible practice, and, for people with eczema and asthma, fulfilled the exposure definition. We collected relevant data from these individuals from their earliest contribution to CPRD through to the earliest date of: study end (July 2020), death if applicable, last data collection or transfer out of an eligible practice.

Eczema and asthma

We included people with eczema at any time up to December 2018 (who may also have asthma) based on an existing validated algorithm requiring at least one diagnostic code for eczema and at least two records for eczema therapies recorded on separate days. We excluded people with a single eczema diagnostic code who failed to meet our eczema algorithm from this study. Similarly, we included people with asthma (who may also have eczema) based on ever having a morbidity code recorded for an asthma diagnosis in primary care. There was no additional requirement to have received asthma therapies. Although eczema and asthma are relapsing and remitting diseases, individuals were classified as having them in this study if they ever met this definition, regardless of recency.

Matched controls

We matched each individual with eczema or asthma to up to five controls, with replacement, on age (within 5 years), sex and general practice from the relevant populations in CPRD GOLD. We used a matching period of 2016-18 and matched individuals on the latest of three matching dates - June 1, 2016, 2017 or 2018 - to attempt to ensure similar follow-up time between exposed individuals and their controls (**Supplementary Figure 1**). Full details of study participant identification are available in **Supplementary Figure 2**.

Supplementary Note 2. Methods

We performed three analysis steps to move from individual level data on Read chapters to a dissimilarity matrix which was used to derive the networks and hierarchical dendrograms presented in this study. Here we describe these steps in more details:

1. Linear mixed-effects logistic regression to estimate association between two Read chapters
2. General linear hypothesis to derive Jaccard distance at specified covariate values
3. Hierarchical cluster analysis and undirected network analysis

Linear mixed-effects logistic regression:

We used a generalised linear mixed model (GLMM) to estimate the probability of recording an event in both Read chapters for all combinations of the 19 chapters included in this study (hence 171 regression models). For each regression let $y_{iq} = 1$ when an individual patient i from practice q records events in both Read chapters being modeled. Then the regression for each pair of Read chapters in matrix notation is as follows:

$$y = X\beta + Zu + \epsilon \qquad (1)$$

Where y is an N×1 vector of binary outcomes where N is the total number of individuals in the model and y = 1 if there are records from both chapters and y = 0 if there is only a record from one chapter (Patients with no record from either chapter were removed for each regression). Then X is an N×3 matrix of the fixed effect predictor variables; exposure status (0 = unexposed, 1 = exposed), age at matching date (centred on age 50), and duration of follow up following the first event (centred on 5 years of follow up). The vector **β** is then then the vector of fixed-effects regression coefficients. Z is the N×q design matrix for the q random effects corresponding to the unique GP practices contributing patients to the corresponding regression. The vector u then captures the random effect and is an N×1 column vector of the residuals. Since patients without a record in either Read chapter under study could not contribute to the likelihood, the size of each regression was different, with varying numbers of individual patients N and practices q. We
used the glmer function from the lme4 package in R:

```
mf <- paste("y ~ ca + age50 + exy5 + mu")
m1r <- glmer(as.formula(paste(mf,"+ (1|pr)")), data=DD, nAGQ=0, family=binomial())
```

Where *y* is the outcome for patient i in practice q, and pr is the practice identifier for q. The fixed effects are estimated for the variables *ca*, a binary indicator for exposure status; *age50*, age at study entry centred on 50; *exy5*, follow up after the first event centred on 5 years; and *mu*, sex of the participant. We ran the model using a logit link function and set the integer scale (nAGQ) to zero. This improved performance of the model and reduced computational burden but provides a less exact form of parameter estimation for GLMMs by optimising the

random effects and the fixed-effects coefficients in the penalized iteratively reweighted least squares step.

General linear hypothesis

To convert our regression output into probabilities we used a general linear hypothesis at relevant parameters values for the fixed effects in our model. We assumed that age and sex would play an important role in the probability of multimorbidity, as well as exposure status. Therefore we derived separate probabilities at pre-specified ages and for both sexes included in this study, and both exposure status (giving 2 × 2 × 2 = 8 networks for eczema and asthma).

To extract the probability of a record from both Read chapters in the model, we used the glht function from the multcomp package in R. The linear hypothesis we tested for each of the eight evaluations was as follows:

```
K <- c("(Intercept) = 0",
        "(Intercept) + caTRUE = 0",
        "(Intercept) + muTRUE = 0",
        "(Intercept) + muTRUE + caTRUE = 0",
        "(Intercept) - 32*age50 = 0",
        "(Intercept) - 32*age50 + caTRUE = 0",
        "(Intercept) + muTRUE - 32*age50 = 0",
        "(Intercept) + muTRUE - 32*age50 + caTRUE = 0")
```

This evaluated the probability of records in both Read chapters in the model for the following:

- Unexposed men at age 50: `(Intercept) = 0`
- Exposed men at age 50: `(Intercept) + caTRUE = 0`
- Unexposed women at age 50: `(Intercept) + muTRUE = 0`
- Exposed women at age 50: `(Intercept) + muTRUE + caTRUE = 0`
- Unexposed men at age 18: `(Intercept) - 32*age50 = 0`
- Exposed men at age 18: `(Intercept) - 32*age50 + caTRUE = 0`
- Unexposed women at age 18: `(Intercept) + muTRUE - 32*age50 = 0`
- Exposed women at age 18: `(Intercept) + muTRUE - 32*age50 + caTRUE = 0`

This function will return the prediction on a logit scale for the probability p of a record from both Read chapters in the model at the corresponding fixed effects values. To convert this estimate (call it $x = log\left(\frac{p}{1-p}\right)$) to a probability we take:

$$p = exp\left(\frac{x}{1+x}\right)$$

These steps result in an estimated probability for a record from two Read chapters for each of the 171 combinations of chapters and the 8 estimated linear hypothesis. We then converted this into a dissimilarity matrix for the hierarchical cluster analysis.

One important note, since the variable exy5 is not included in these linear hypotheses, the fixed effect of follow up was controlled for at a centred value of 5 years. In other words, these hypotheses test for the probability that a man/woman, exposed/unexposed, 18/50 year-old, would record a Read code in the other chapter within 5 years of a record in the first (for each pair of studied Read chapters).

Hierarchical cluster analysis and undirected network analysis

We constructed a dissimilarity matrix (8 × 190) for the probability of multiple records from each combination of two Read chapters. There were 8 for combinations of the two specified ages (18 and 50 years-old), sexes (male and female) and exposure statuses (exposed and unexposed). There were 190 rows in the matrix for the 171 possible discordant combinations plus the 19 chapters themselves. This dissimilarity matrix was then analysed using the hclust function from the stats package using the complete linkage clustering method. This performs a hierarchical cluster analysis on a set of dissimilarities for the 19 Read chapters being clustered at each of the 8 combinations of explanatory variable values.

In short, each object is assigned to its own cluster and then the algorithm proceeds iteratively, at each stage joining the two most similar clusters, continuing until there is just a single cluster. At each step the two clusters separated by the "shortest distance" are combined, in our example this is the shortest Jaccard distance (or highest probability of a record from both Read chapters in pairs). The method is an agglomerative scheme that erases rows and columns in the matrix as old clusters are merged into new ones.

The `hclust` generates a hierarchical tree produced by the clustering process. This tree is then used to plot the dendrograms in this study. The undirected network graphs were constructed using the `graph_from_data_frame` function from the igraph package where the node size is determined by the number of recorded events in each specific Read chapter for each combination of age, sex and exposure in our analysis. The edges are determined by the conditional probability of a record in one chapter given a record in another, from the GLMM analysis above

Supplementary Figure 1. Illustration of the study design. We used routinely recorded information up to July 2020 to define records by Read code chapter. Participants were defined as exposed to eczema or asthma according to specific diagnostic criteria using data up to 2016. We then identified a matched control population in the window June 2016-2018 and matched participants to upto five controls on the latest of three matching dates that exposed individuals were eligible for inclusion (June 2016-18).

Figure 1: From transactional data to study implementation*

Individual-patient data is documented as encounters from various sources, including diagnoses/procedures (Dx/Px), drug dispensings (Rx). It is arranged in *calendar time*.

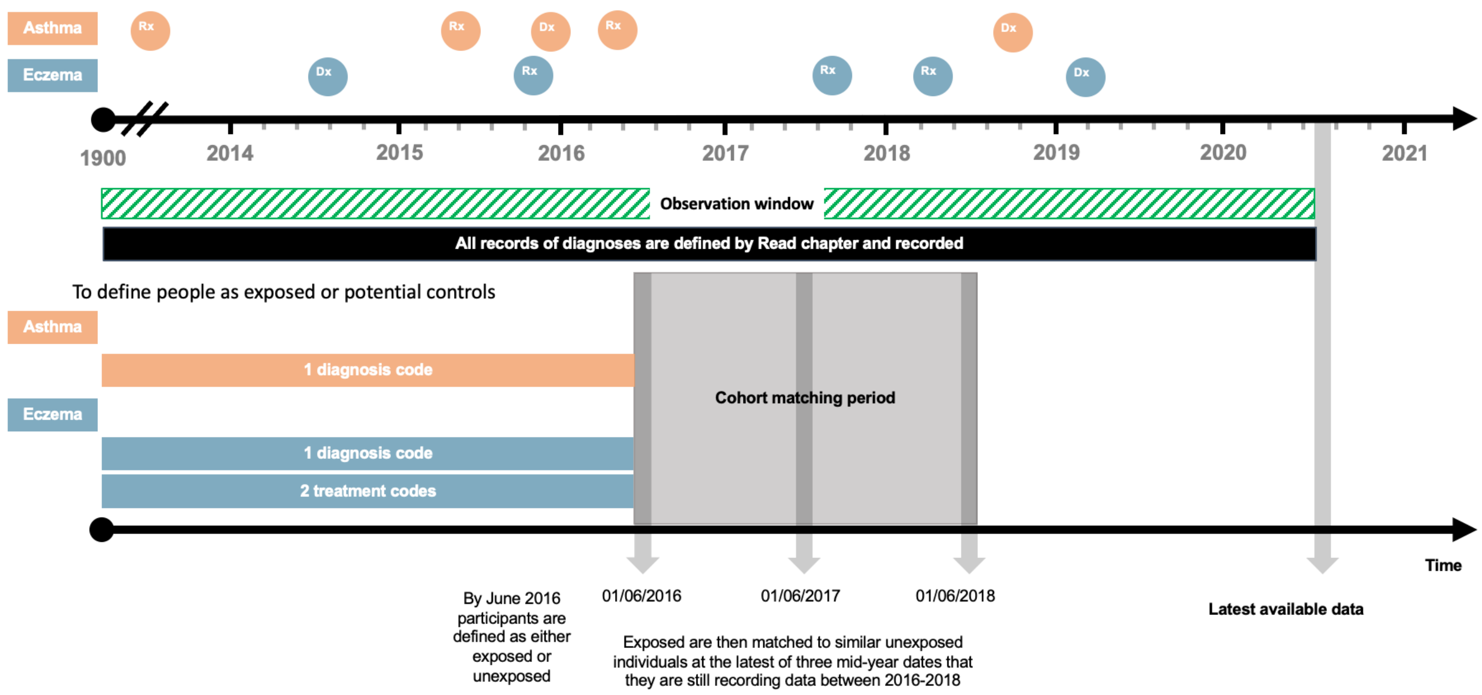

Supplementary Figure 2. Flowchart illustrating selection of the study populations

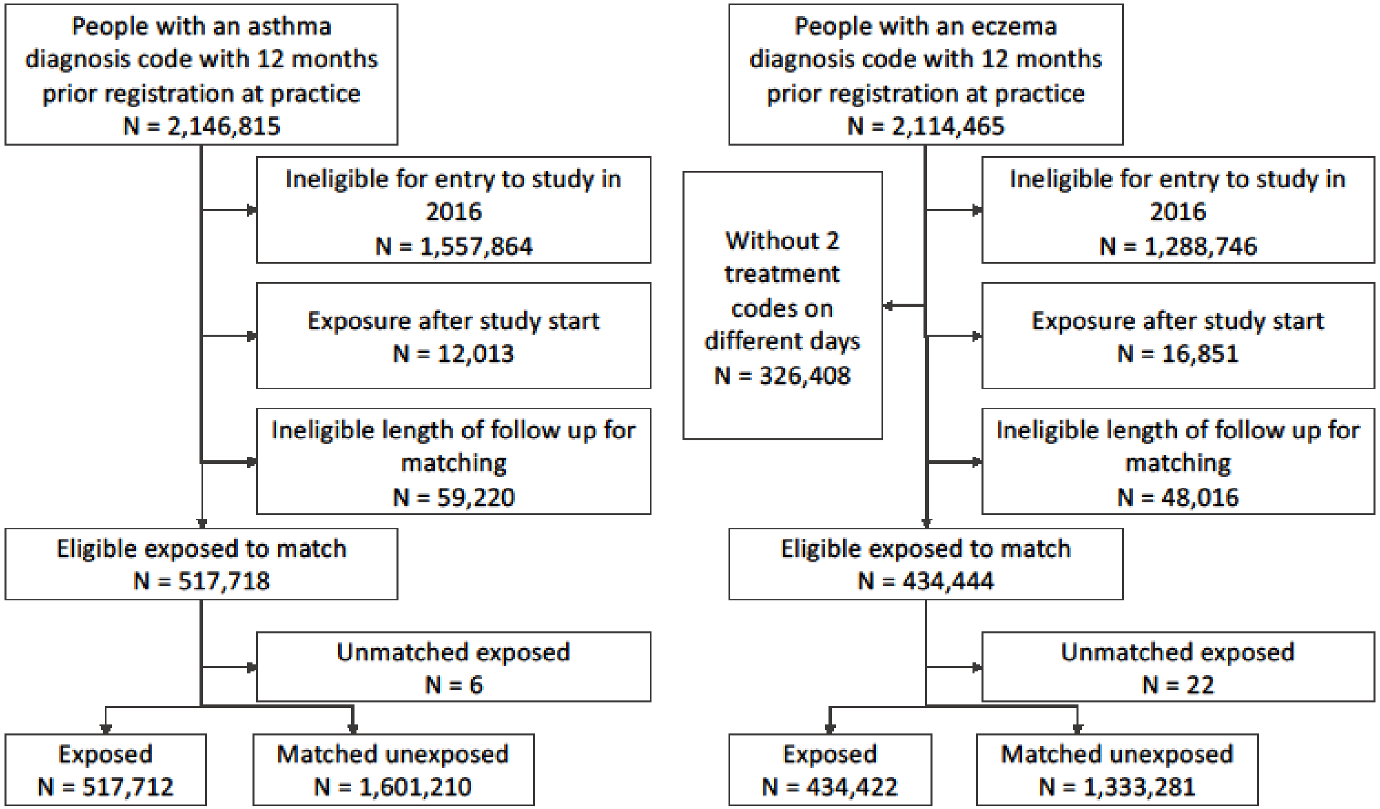

Supplementary Figure 3: Proportion of Read chapter records in the study population: bar chart illustrating the total number of people that ever recorded a diagnostic code by Read chapter (top panel) and the proportion of the study population that ever recorded a diagnostic code in each Read chapter (bottom panel) for exposed individuals and matched controls in the asthma and eczema cohort. The total percentage of people with asthma that recorded a chapter H (respiratory) diagnostic code is slightly less than 100% because administrative codes (Chapters [1-9]) were also used to define asthma (see supplementary methods)

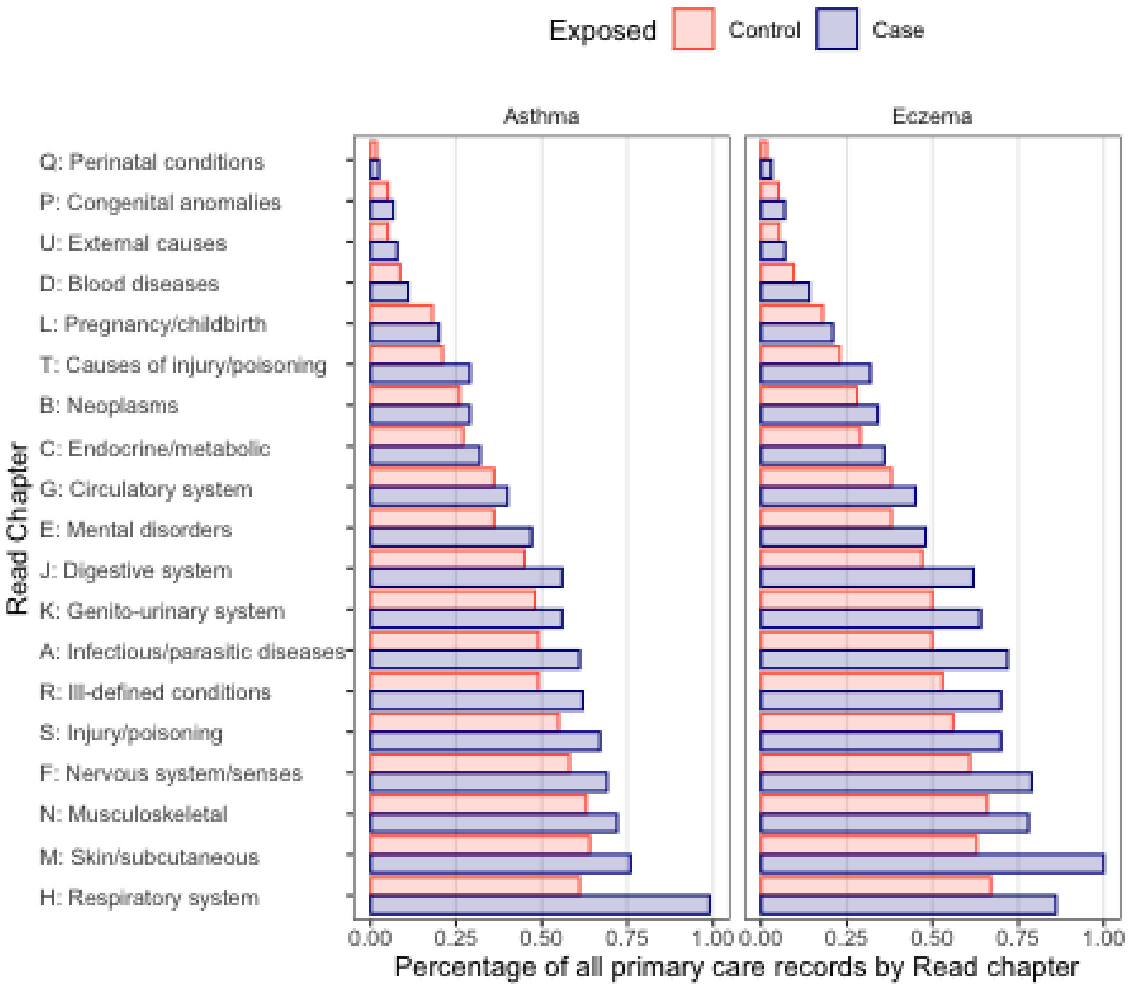

Variable	Exposed	Asthma	Eczema
N	Control	1,601,210	1,333,281
	Case	517,712	434,422

Supplementary Figure 4: Descriptive summary statistics from mixed-effects logistic regression models used to estimate Jaccard indices for asthma clustering analyses. Models were fitted on subpopulations of individuals who had diagnoses recorded from either or both of two Read chapters, with the outcome being a diagnosis from both (see main paper). We considered 19 Read chapters giving 171 pairwise combinations. The 171 data points in the histograms are summary statistics of the relevant subpopulations.

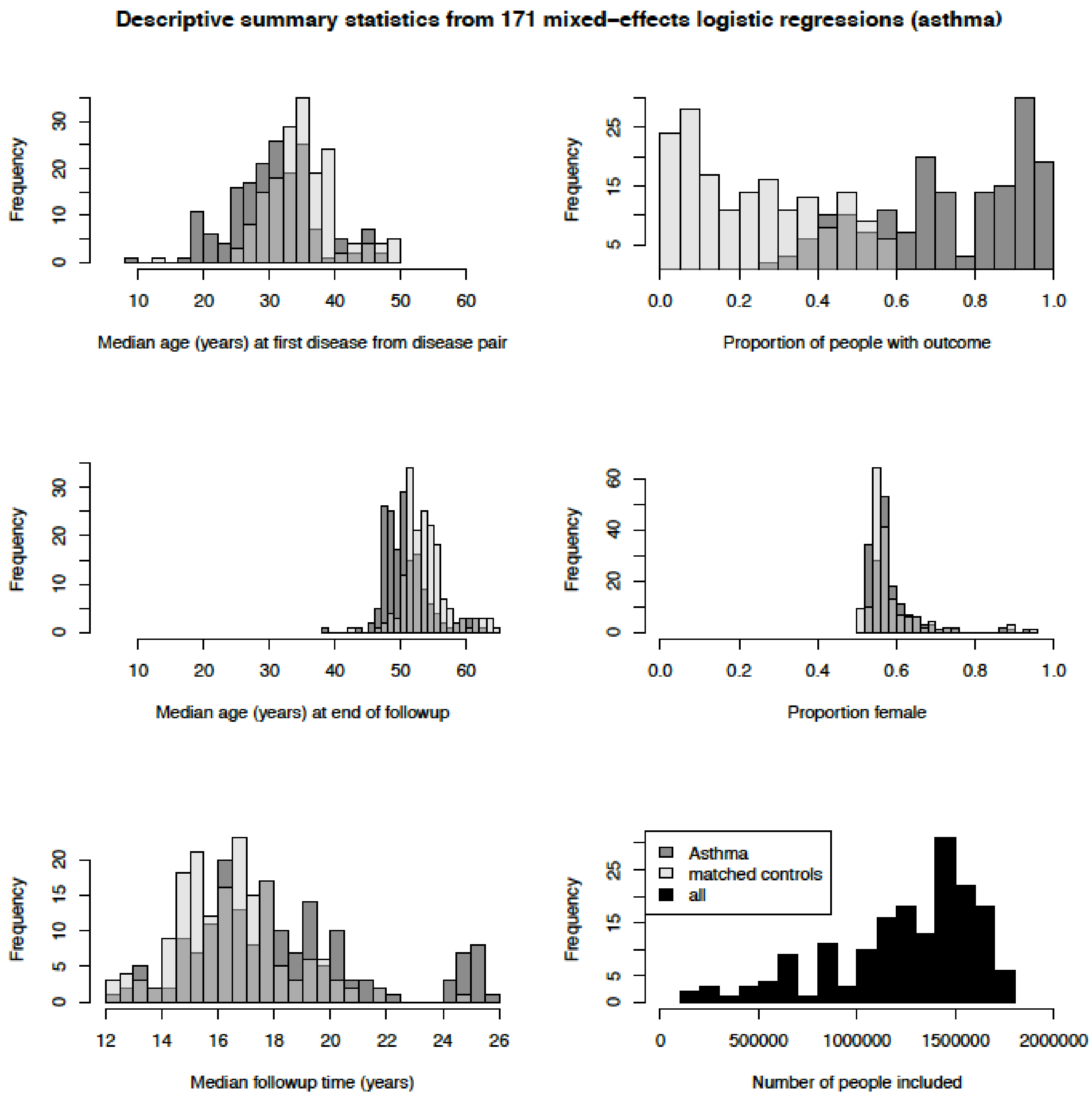

Supplementary Figure 5: Descriptive summary statistics from mixed-effects logistic regression models used to estimate Jaccard indices for eczema clustering analyses. Models were fitted on subpopulations of individuals who had diagnoses recorded from either or both of two Read chapters, with the outcome being a diagnosis from both (see main paper). We considered 19 Read chapters giving 171 pairwise combinations. The 171 data points in the histograms are summary statistics of the relevant subpopulations.

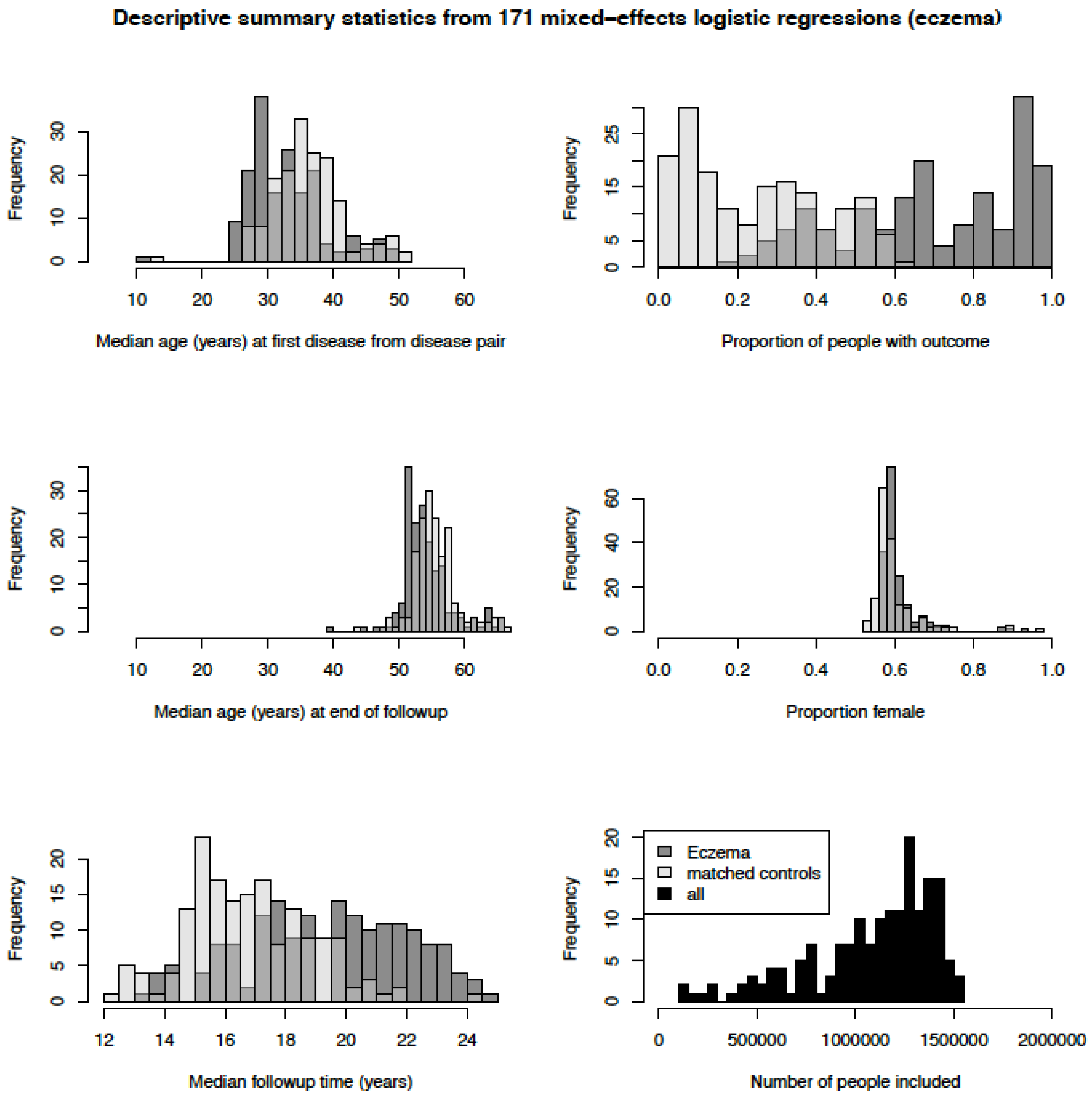

Supplementary Figure 6. Detailed dendrograms (A, B) and undirected networks (C, D) of Read code chapter clusters for MEN aged 18 with and without eczema with indications at (dendrograms) or using a cut point of (networks) 30% probability of co-occurrence. All probabilities are calculated at 5 years' followup from the first chapter recording. Clusters of chapters with a greater than 30% probability of co-occurrence are shown in color (Blue, eczema. Red, matched controls); labels for chapters including eczema and asthma are indicated in red. Thicker edges (networks) indicate stronger probabilities of co-occurrence.

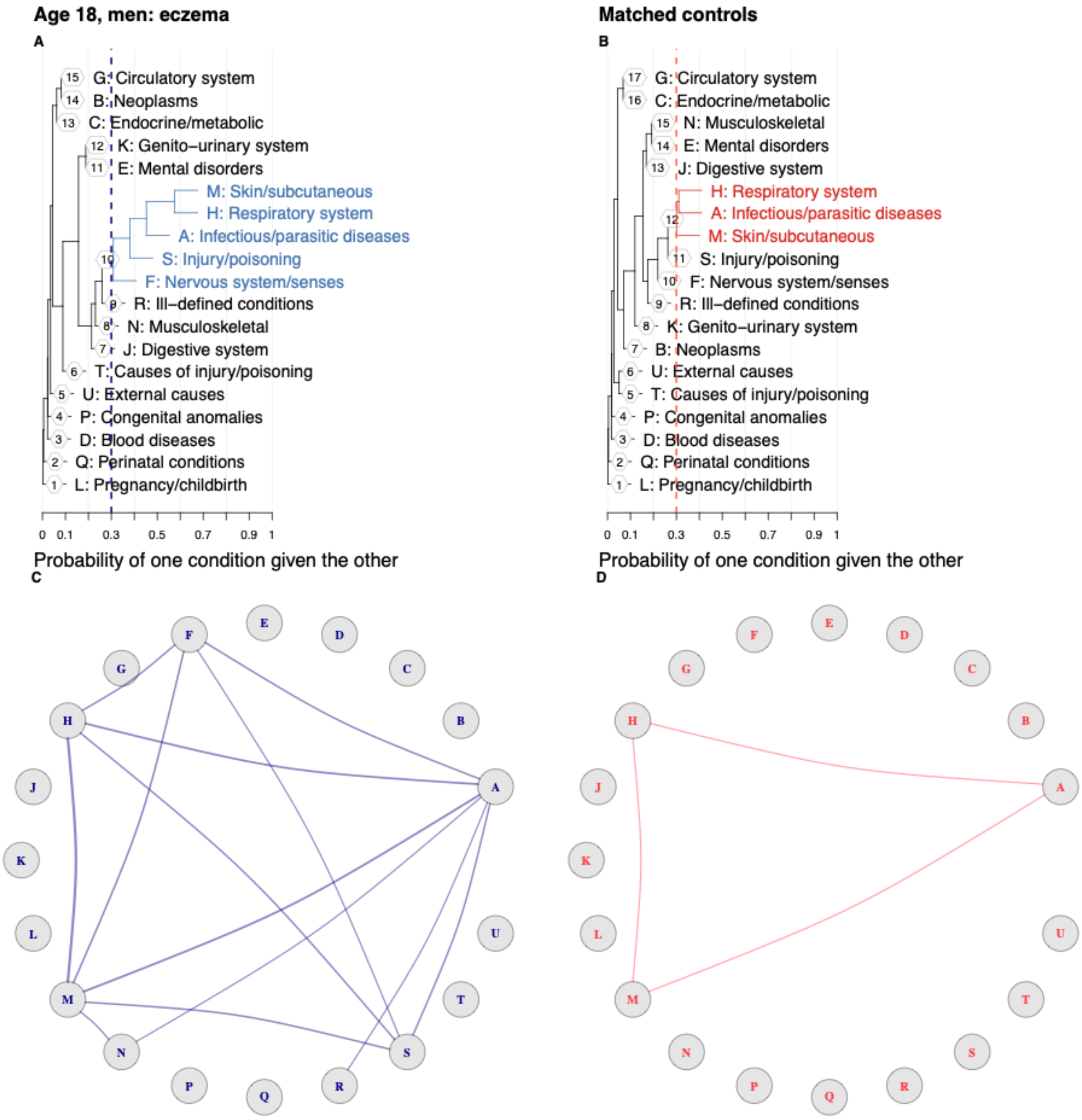

Supplementary Figure 7. Detailed dendrograms (A, B) and undirected networks (C, D) of Read code chapter clusters for WOMEN aged 18 with and without eczema with indications at (dendrograms) or using a cut point of (networks) 30% probability of co-occurrence. All probabilities are calculated at 5 years' followup from the first chapter recording. Clusters of chapters with a greater than 30% probability of co-occurrence are shown in color (Blue, eczema. Red, matched controls); labels for chapters including eczema and asthma are indicated in red. Thicker edges (networks) indicate stronger probabilities of co-occurrence.

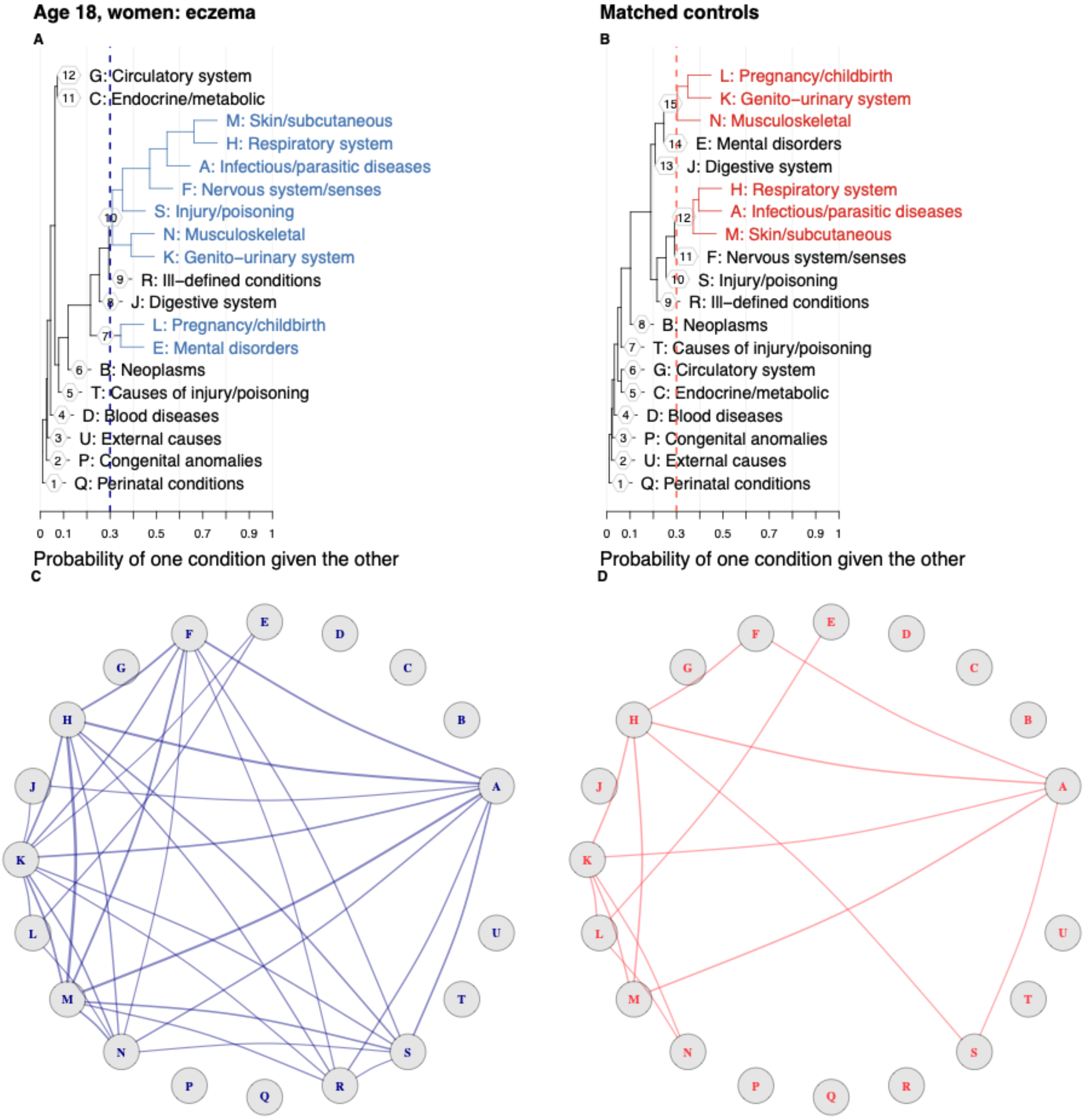

Supplementary Figure 8. Detailed dendrograms (A, B) and undirected networks (C, D) of Read code chapter clusters for MEN aged 50 with and without eczema with indications at (dendrograms) or using a cut point of (networks) 30% probability of co-occurrence. All probabilities are calculated at 5 years' followup from the first chapter recording. Clusters of chapters with a greater than 30% probability of co-occurrence are shown in color (Blue, eczema. Red, matched controls); labels for chapters including eczema and asthma are indicated in red. Thicker edges (networks) indicate stronger probabilities of co-occurrence.

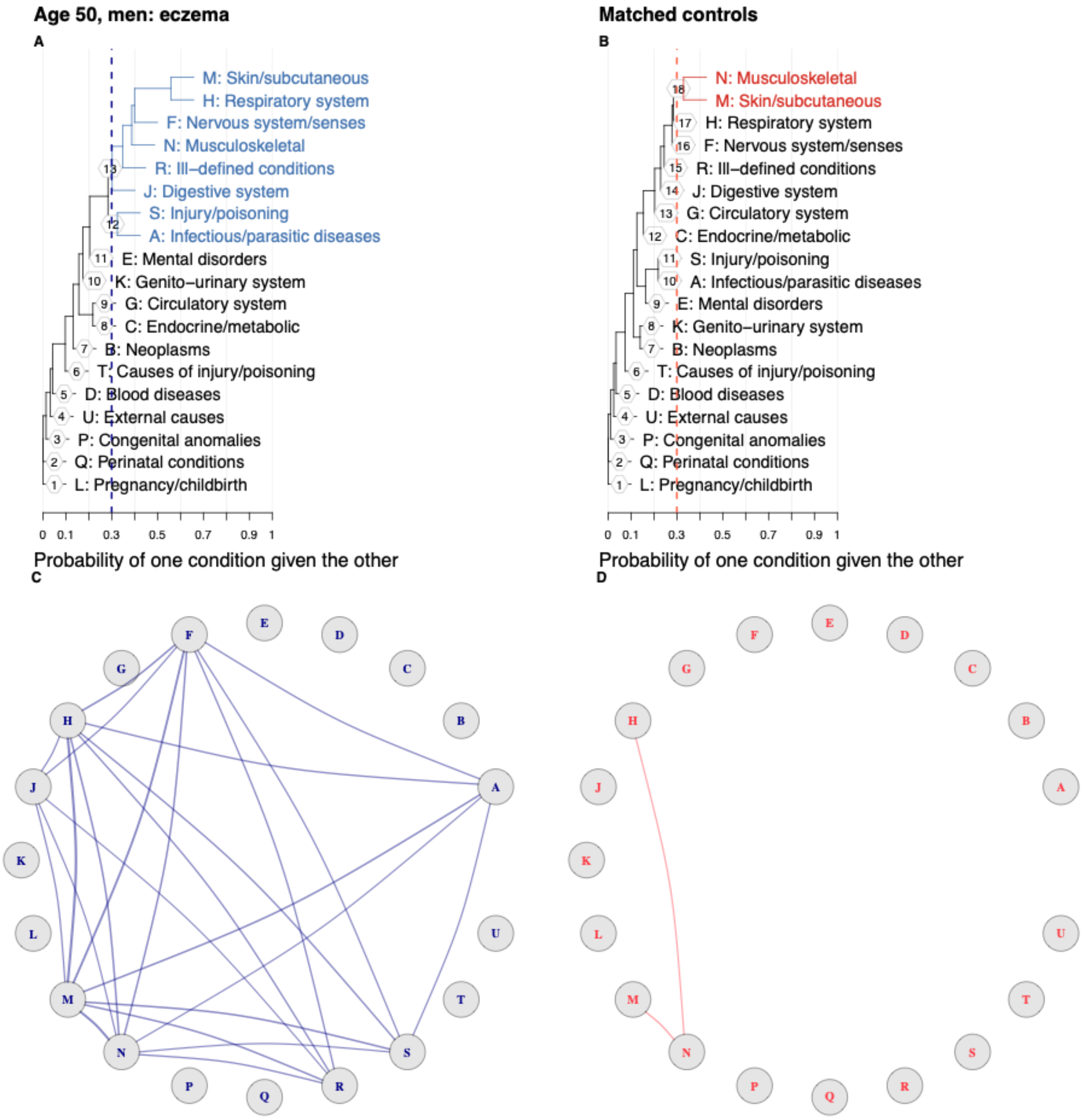

Supplementary Figure 9. Detailed dendrograms (A, B) and undirected networks (C, D) of Read code chapter clusters for WOMEN aged 50 with and without eczema with indications at (dendrograms) or using a cut point of (networks) 30% probability of co-occurrence. All probabilities are calculated at 5 years' followup from the first chapter recording. Clusters of chapters with a greater than 30% probability of co-occurrence are shown in color (Blue, eczema. Red, matched controls); labels for chapters including eczema and asthma are indicated in red. Thicker edges (networks) indicate stronger probabilities of co-occurrence.

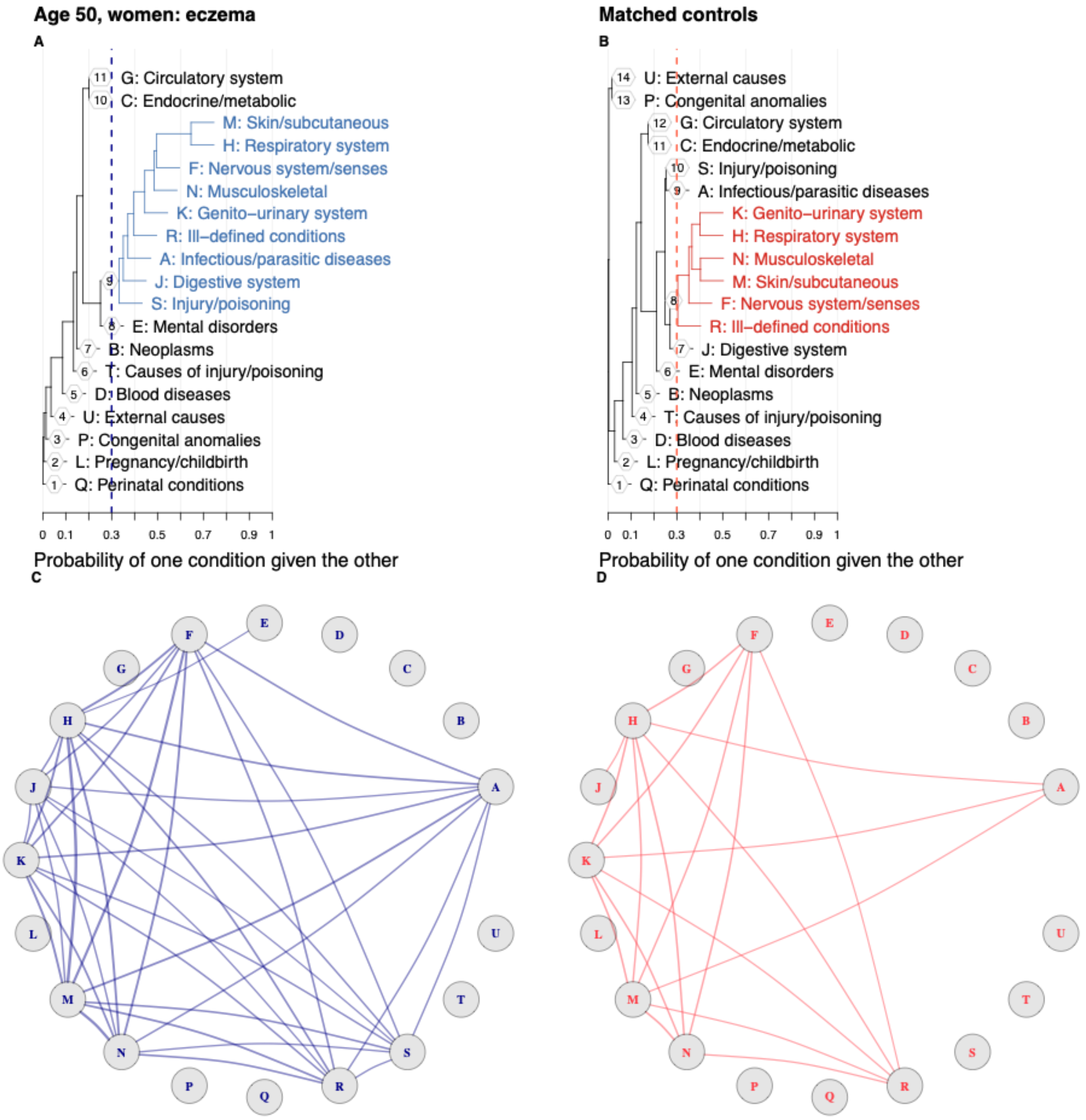

Supplementary Figure 10. Detailed dendrograms (A, B) and undirected networks (C, D) of Read code chapter clusters for MEN aged 18 with and without asthma with indications at (dendrograms) or using a cut point of (networks) 30% probability of co-occurrence. All probabilities are calculated at 5 years' followup from the first chapter recording. Clusters of chapters with a greater than 30% probability of co-occurrence are shown in color (Blue, asthma. Red, matched controls); labels for chapters including eczema and asthma are indicated in red. Thicker edges (networks) indicate stronger probabilities of co-occurrence.

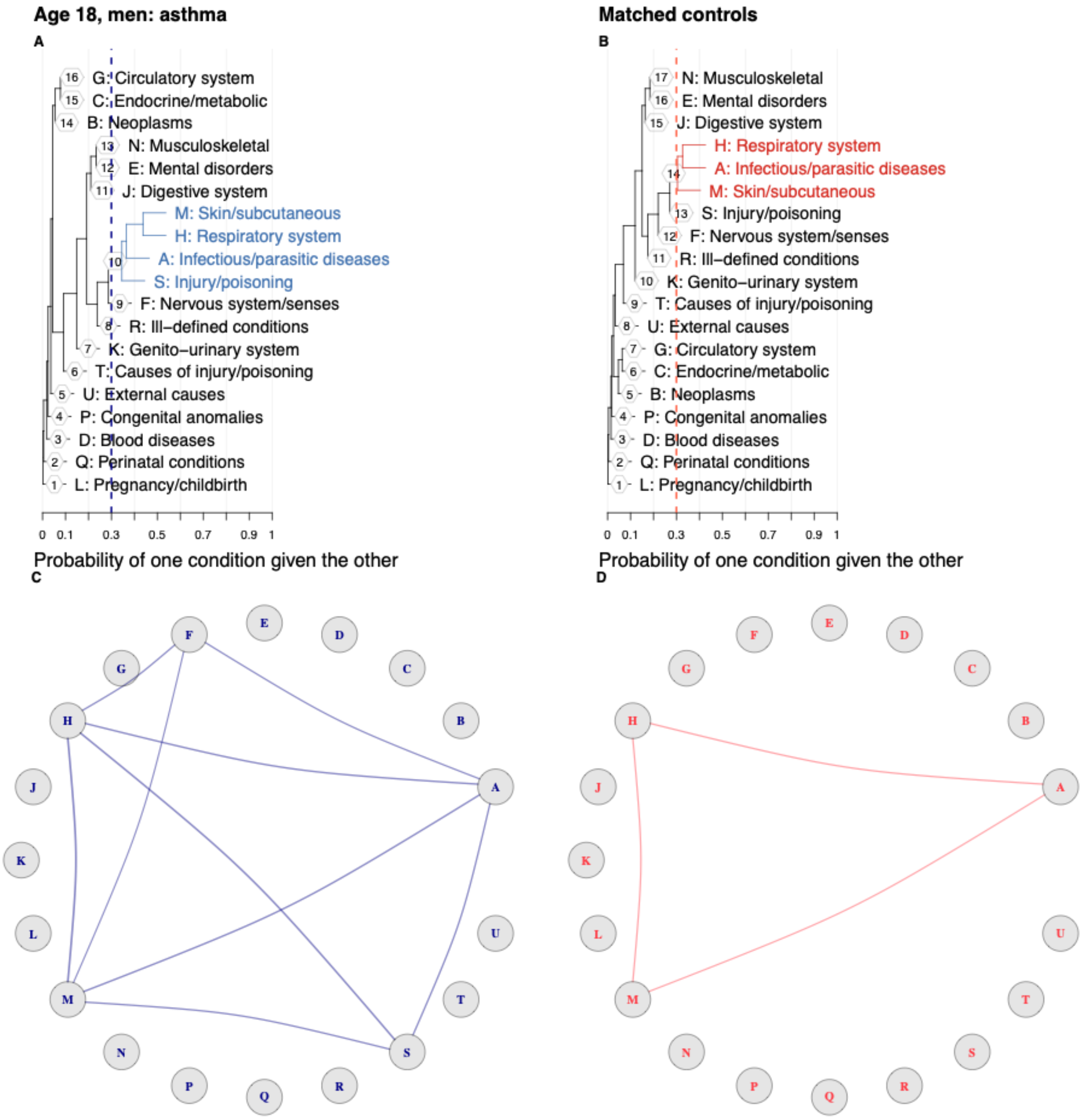

Supplementary Figure 11. Detailed dendrograms (A, B) and undirected networks (C, D) of Read code chapter clusters for WOMEN aged 18 with and without asthma with indications at (dendrograms) or using a cut point of (networks) 30% probability of co-occurrence. All probabilities are calculated at 5 years' followup from the first chapter recording. Clusters of chapters with a greater than 30% probability of co-occurrence are shown in color (Blue, asthma. Red, matched controls); labels for chapters including eczema and asthma are indicated in red. Thicker edges (networks) indicate stronger probabilities of co-occurrence.

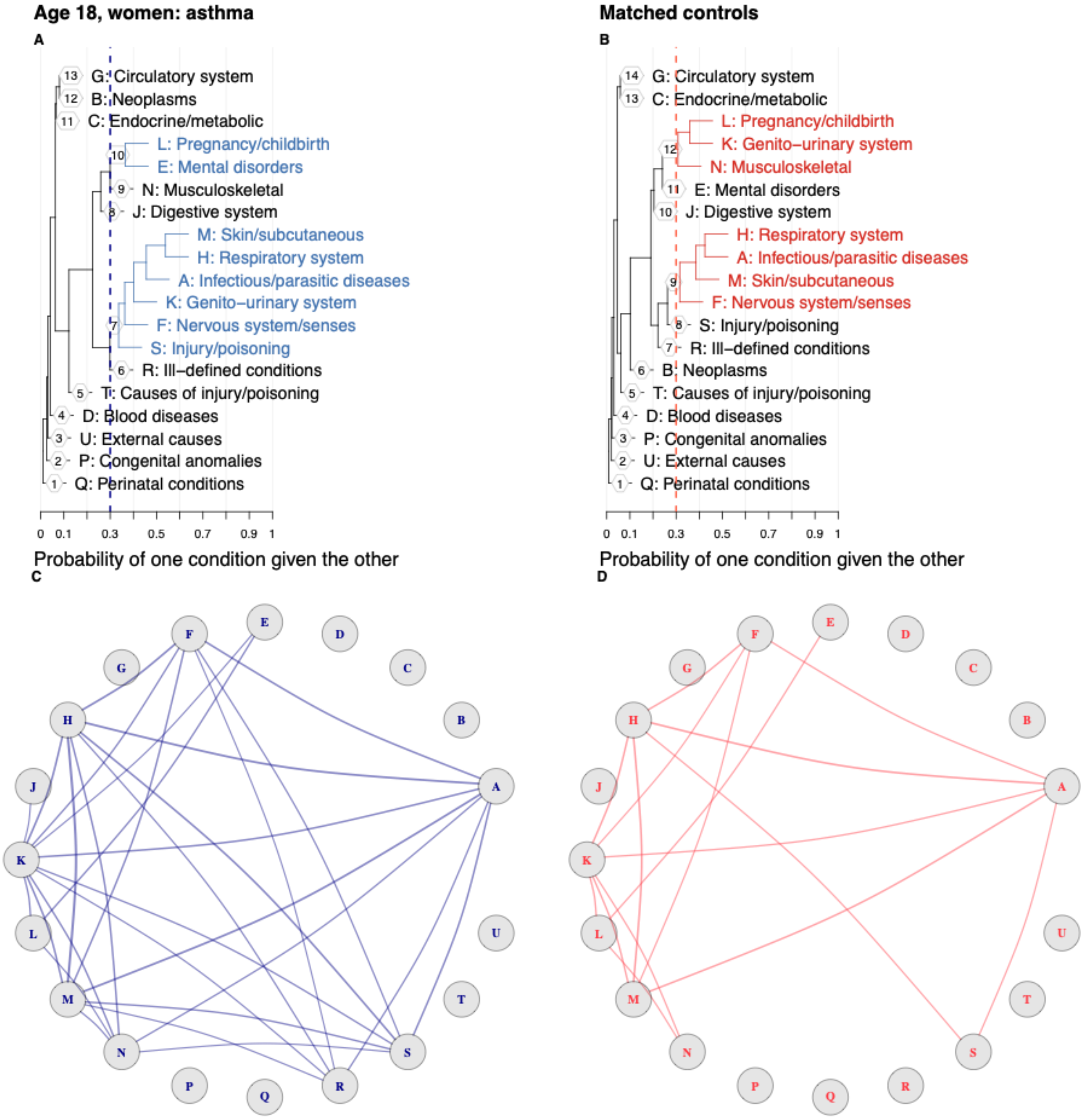

Supplementary Figure 12. Detailed dendrograms (A, B) and undirected networks (C, D) of Read code chapter clusters for MEN aged 50 with and without asthma with indications at (dendrograms) or using a cut point of (networks) 30% probability of co-occurrence. All probabilities are calculated at 5 years' followup from the first chapter recording. Clusters of chapters with a greater than 30% probability of co-occurrence are shown in color (Blue, asthma. Red, matched controls); labels for chapters including eczema and asthma are indicated in red. Thicker edges (networks) indicate stronger probabilities of co-occurrence.

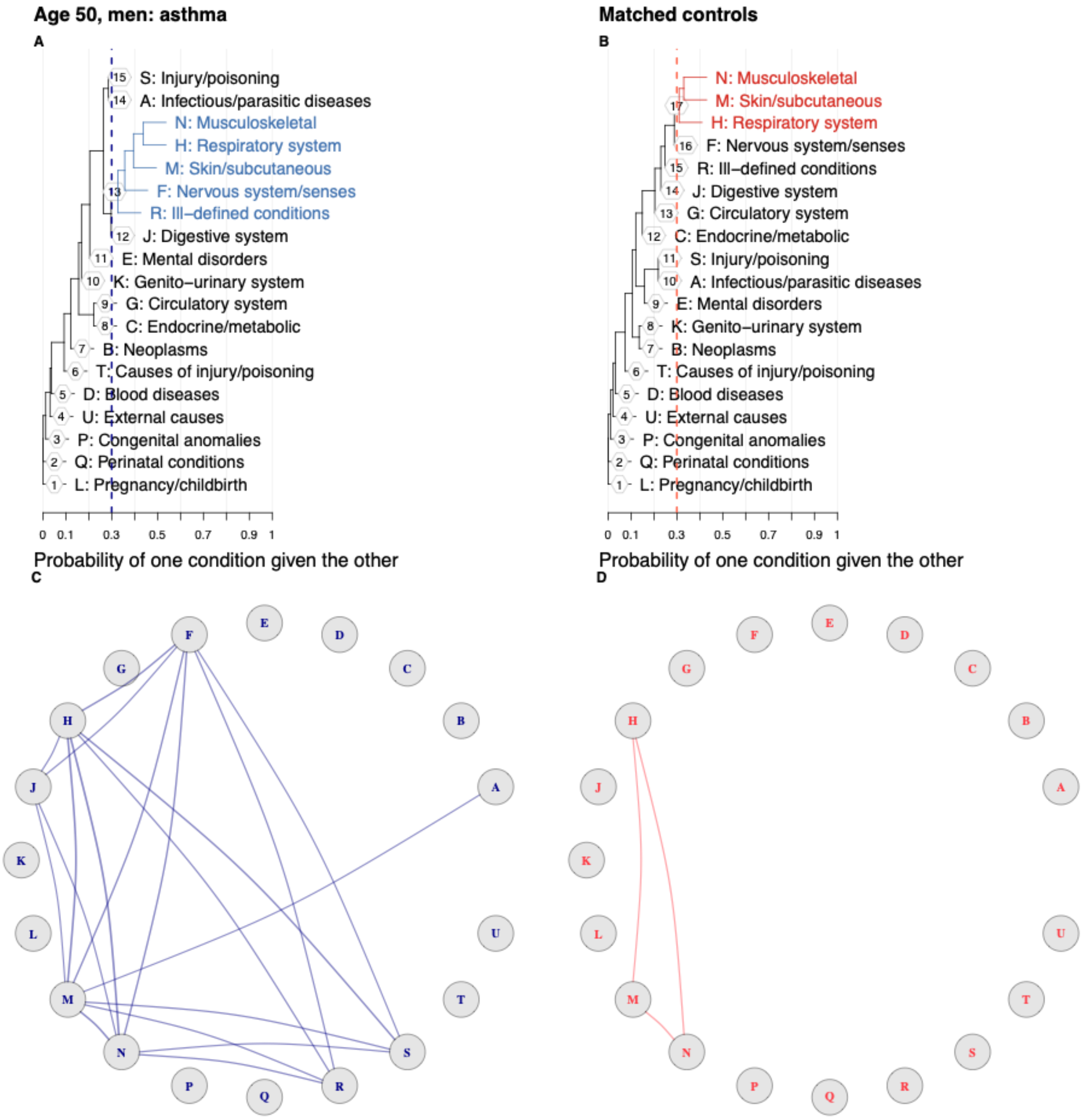

Supplementary Figure 13. Detailed dendrograms (A, B) and undirected networks (C, D) of Read code chapter clusters for WOMEN aged 50 with and without asthma with indications at (dendrograms) or using a cut point of (networks) 30% probability of co-occurrence. All probabilities are calculated at 5 years' followup from the first chapter recording. Clusters of chapters with a greater than 30% probability of co-occurrence are shown in color (Blue, asthma. Red, matched controls); labels for chapters including eczema and asthma are indicated in red. Thicker edges (networks) indicate stronger probabilities of co-occurrence.

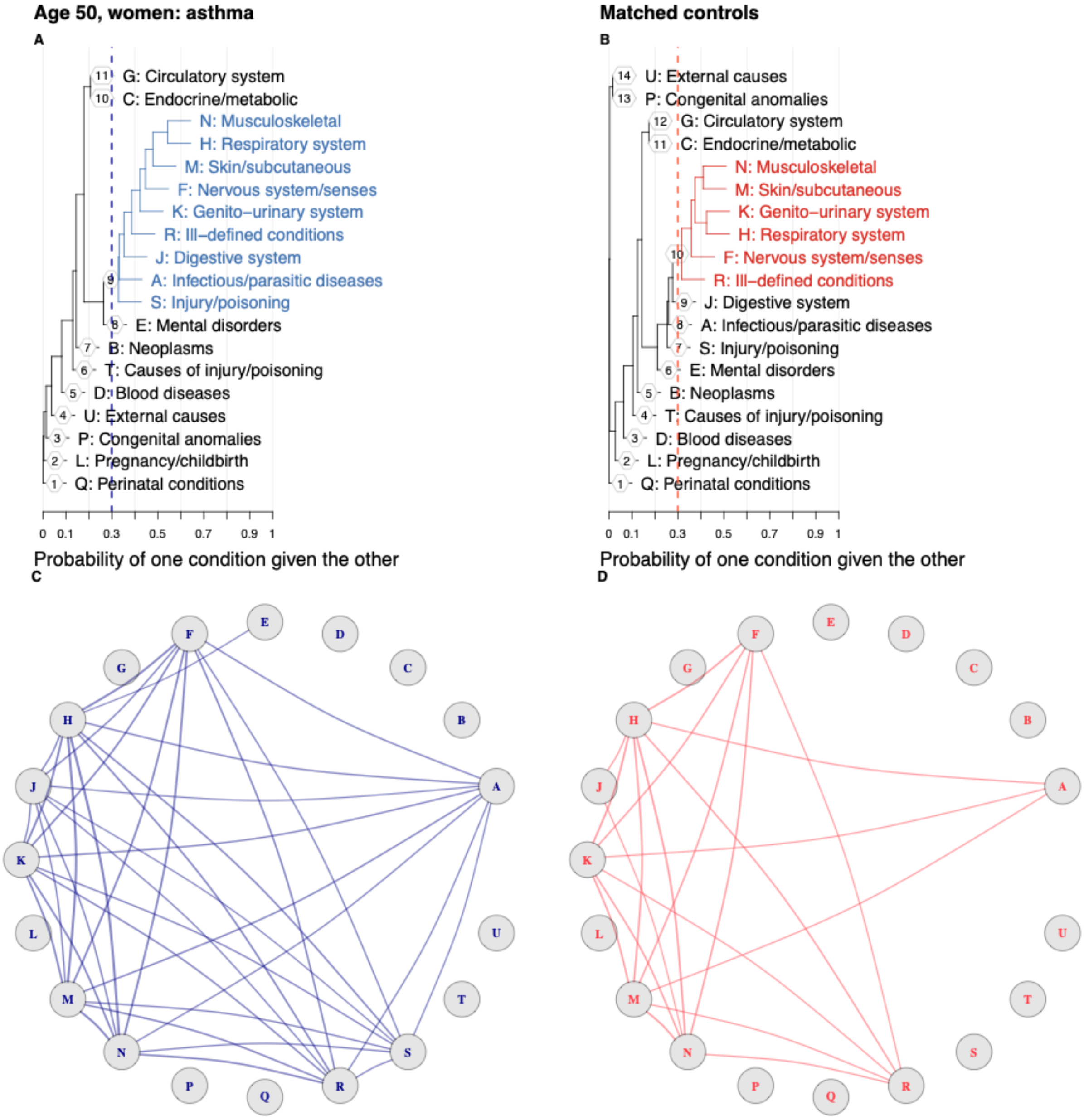

Supplementary Figure 14(A). Asthma cluster differences with 1, 5 and 10 years of followup

Age 18, men: matched controls, fu=1 years (complete linkage)

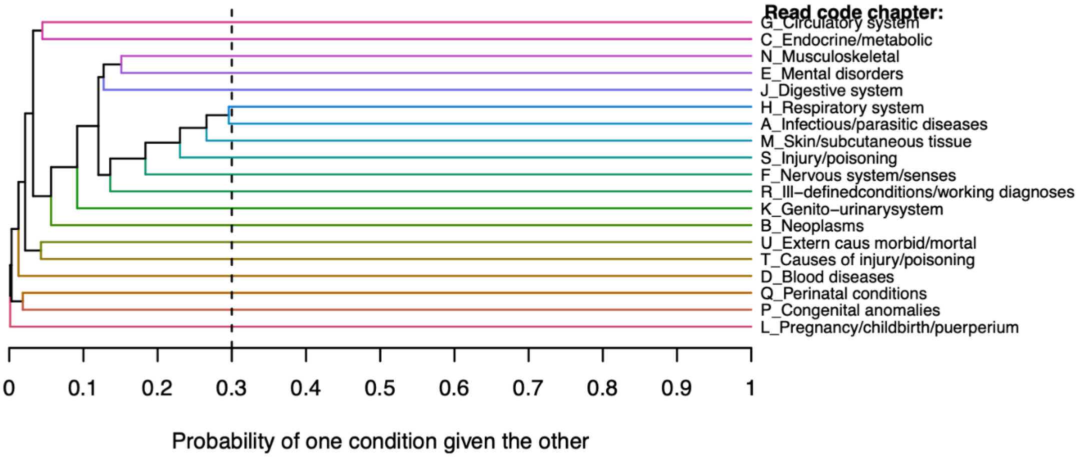

Age 18, men: matched controls, fu=5 years (complete linkage)

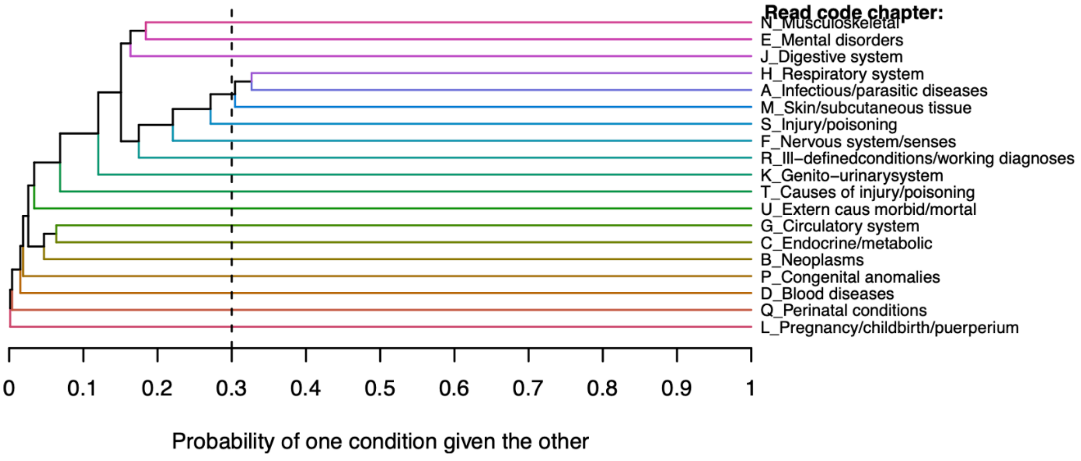

Age 18, men: matched controls, fu=10 years (complete linkage)

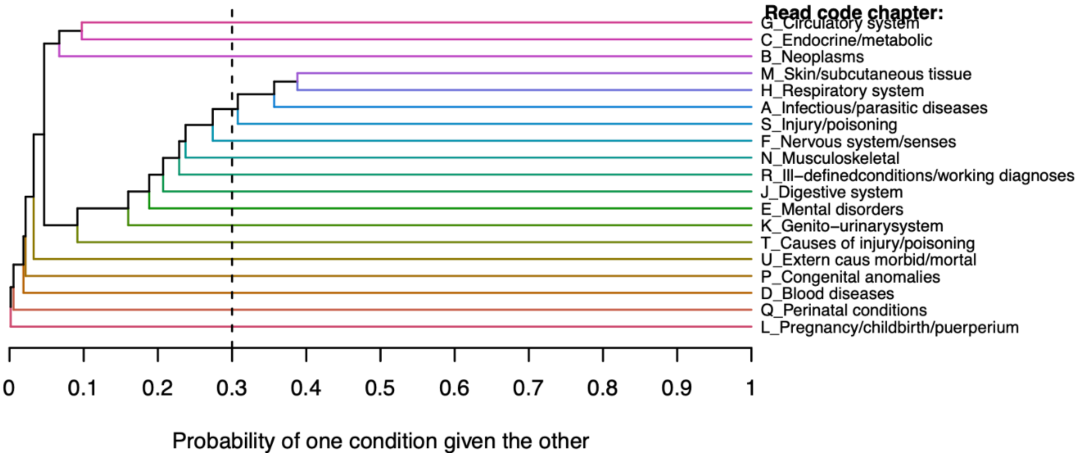

Supplementary Figure 14(B). Asthma cluster differences with 1, 5 and 10 years of followup

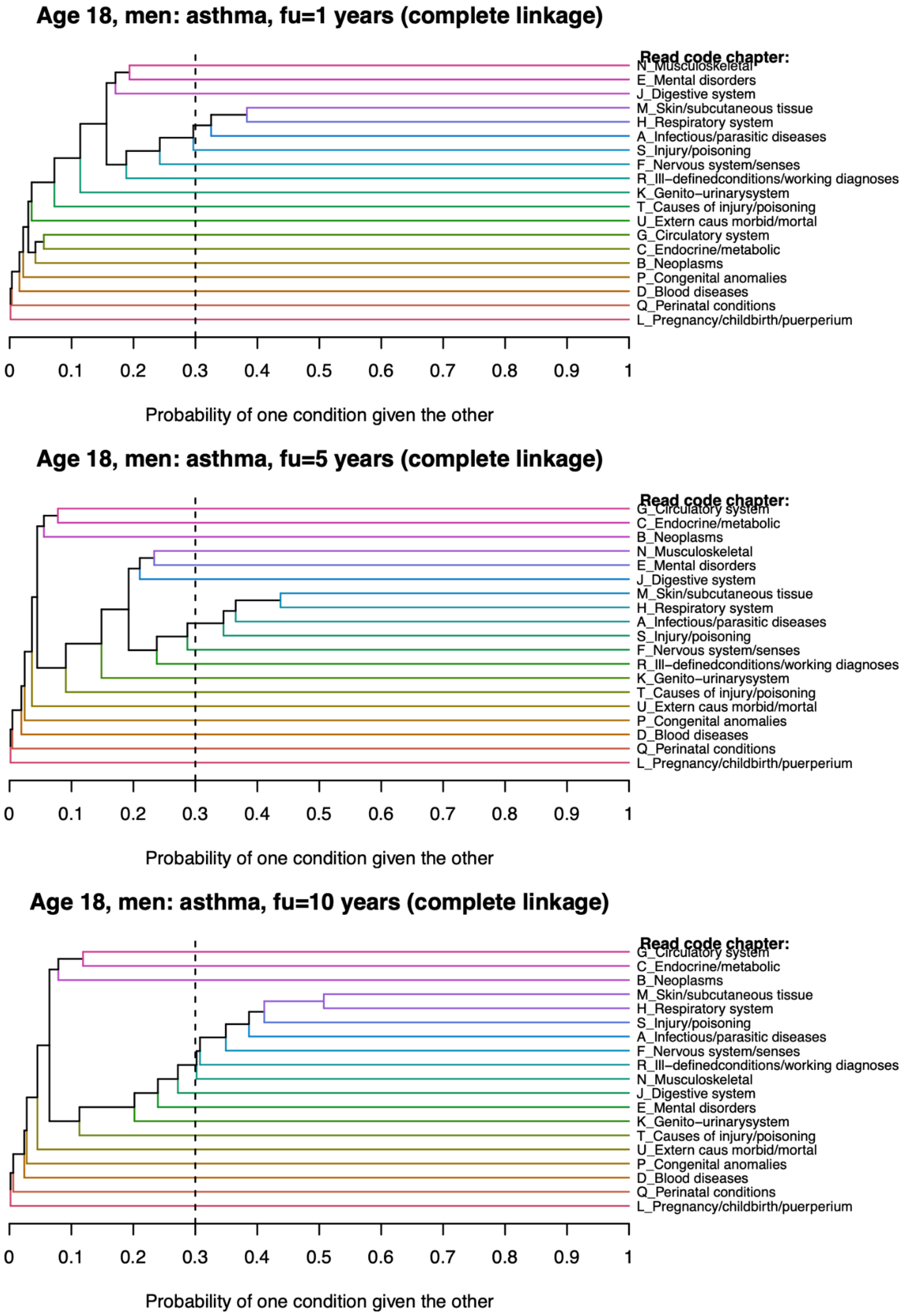

Supplementary Figure 14(C). Asthma cluster differences with 1, 5 and 10 years of

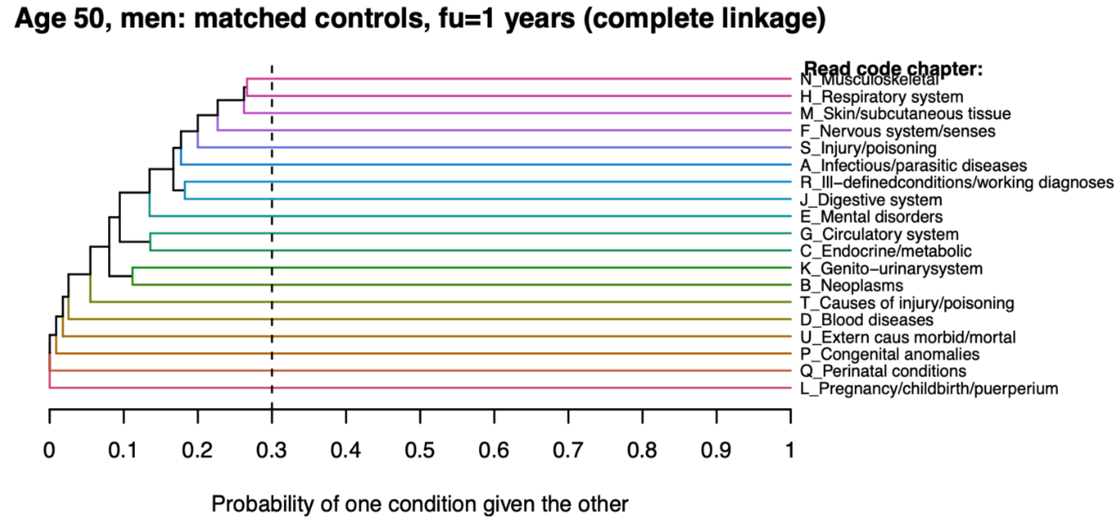

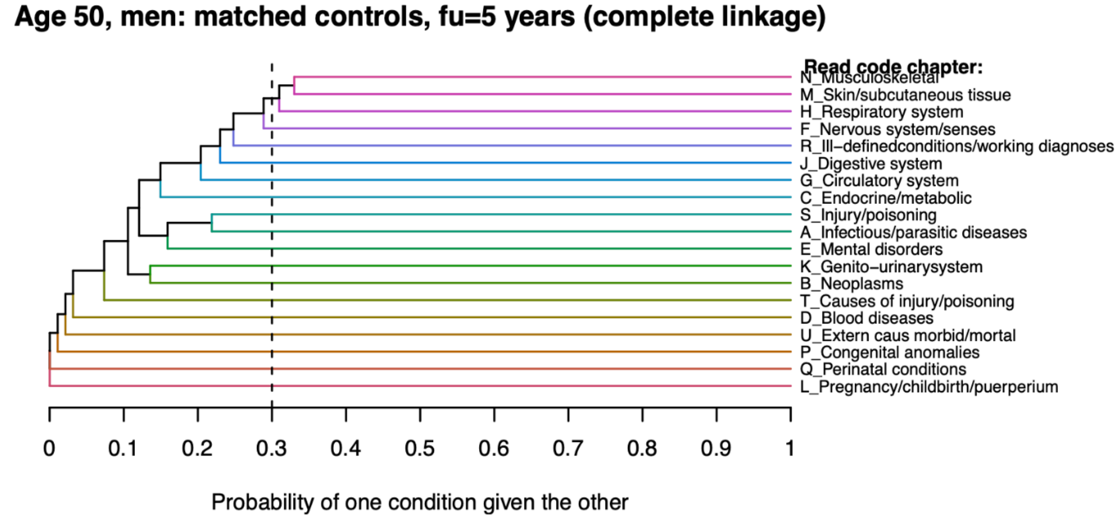

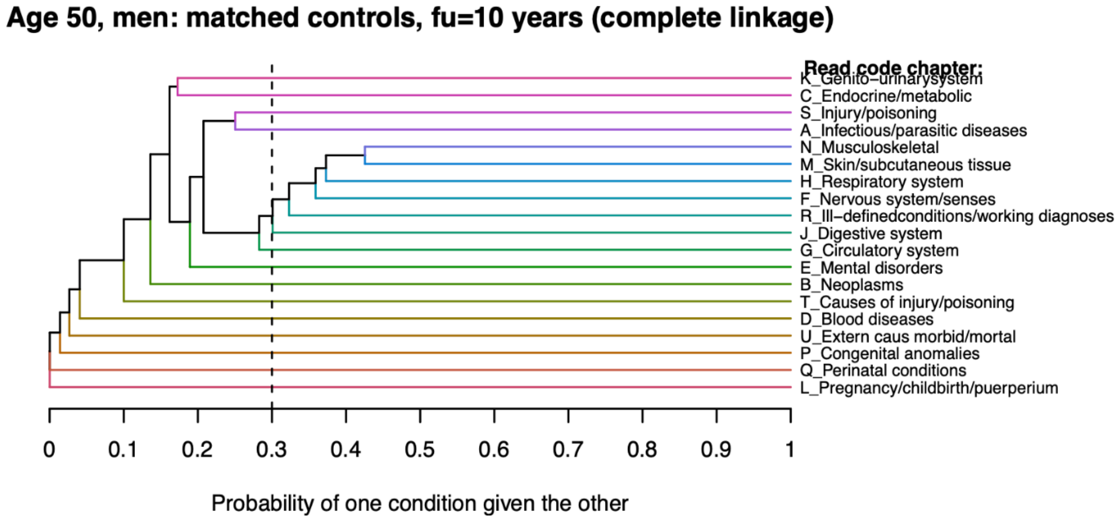

Supplementary Figure 14(D). Asthma cluster differences with 1, 5 and 10 years of followup

Age 50, men: asthma, fu=1 years (complete linkage)

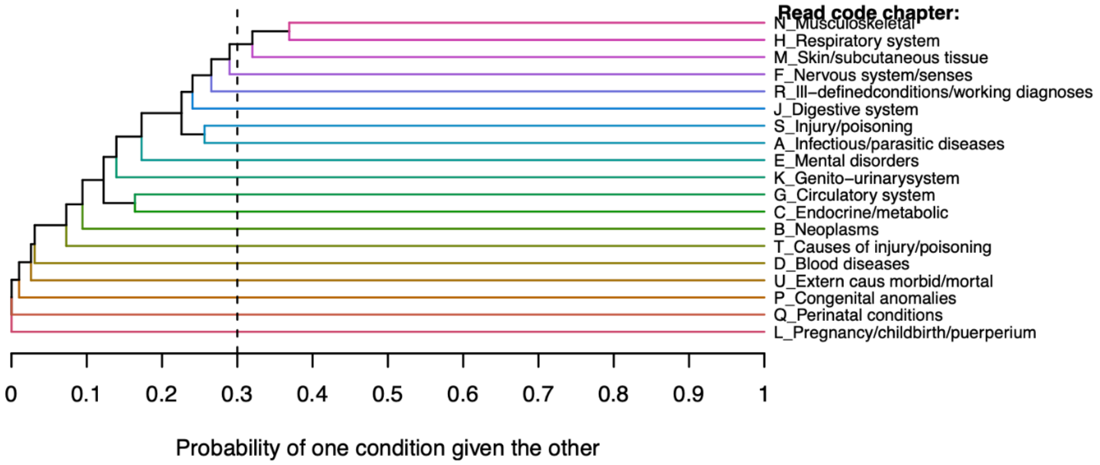

Age 50, men: asthma, fu=5 years (complete linkage)

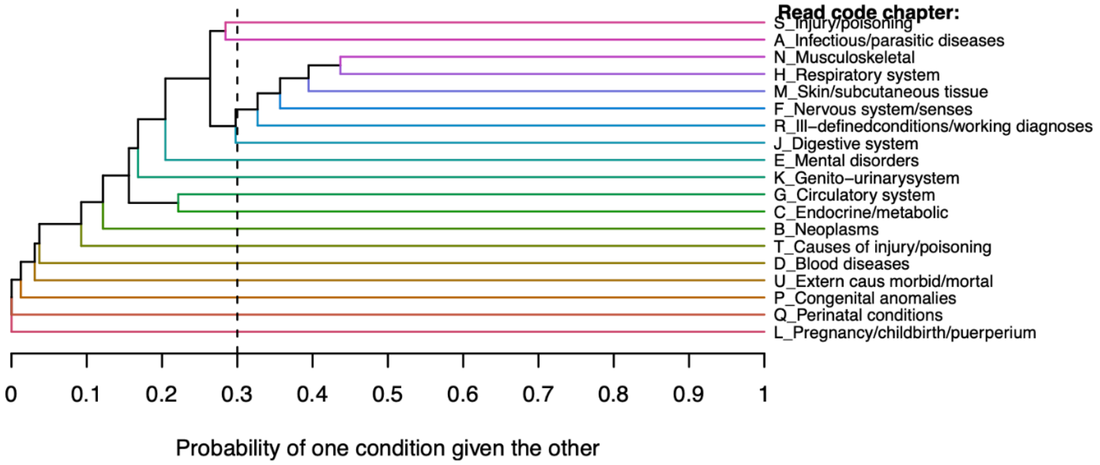

Age 50, men: asthma, fu=10 years (complete linkage)

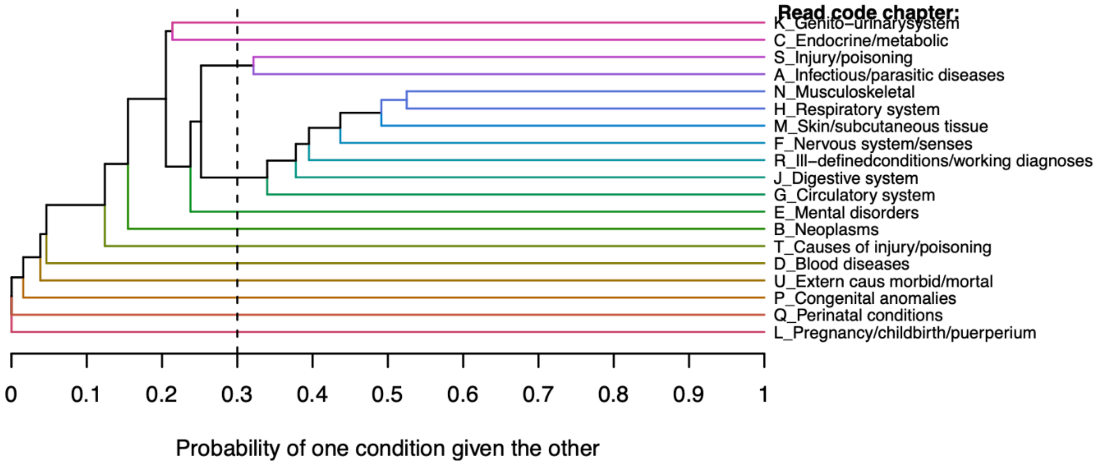

Supplementary Figure 14(E). Asthma cluster differences with 1, 5 and 10 years of

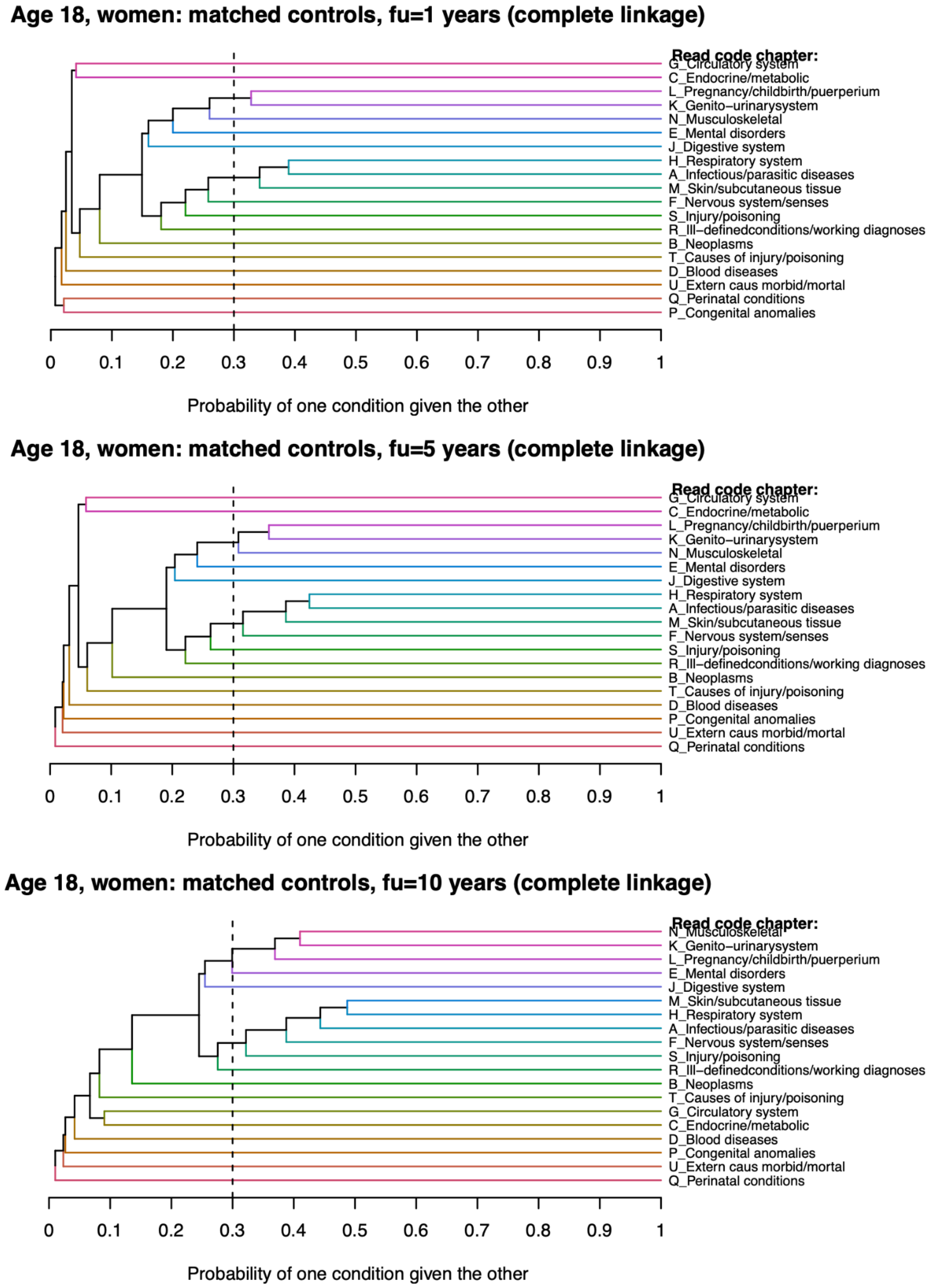

Supplementary Figure 14(F). Asthma cluster differences with 1, 5 and 10 years of followup

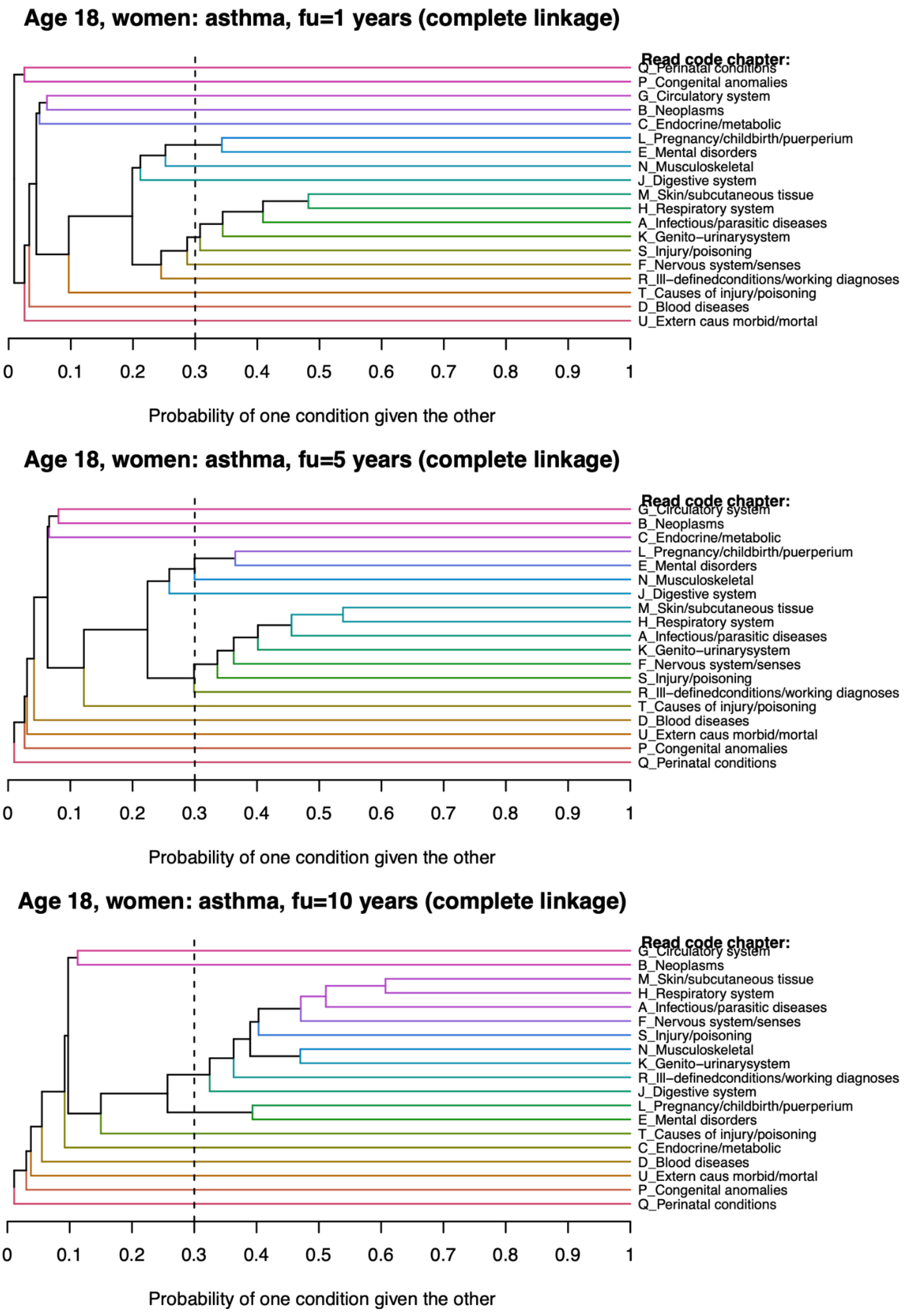

Supplementary Figure 14(G). Asthma cluster differences with 1, 5 and 10 years of

Age 50, women: matched controls, fu=1 years (complete linkage)

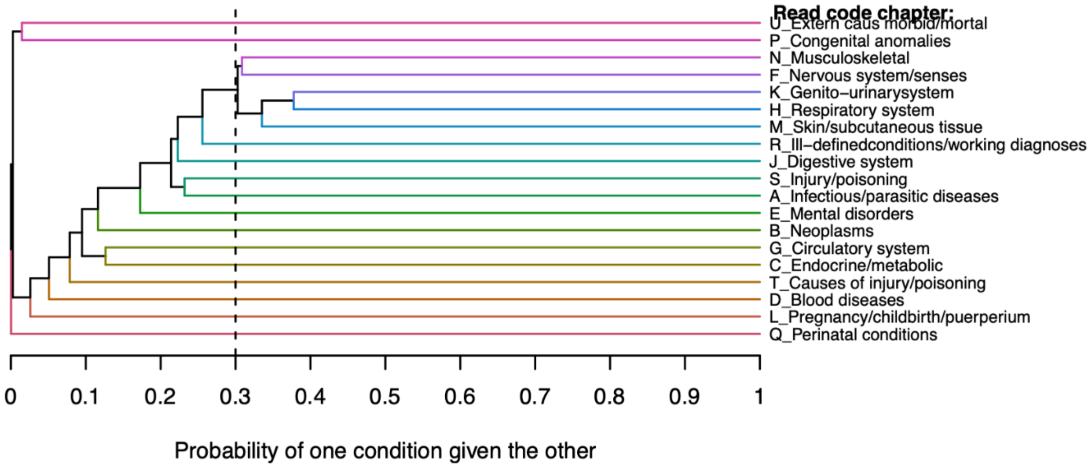

Age 50, women: matched controls, fu=5 years (complete linkage)

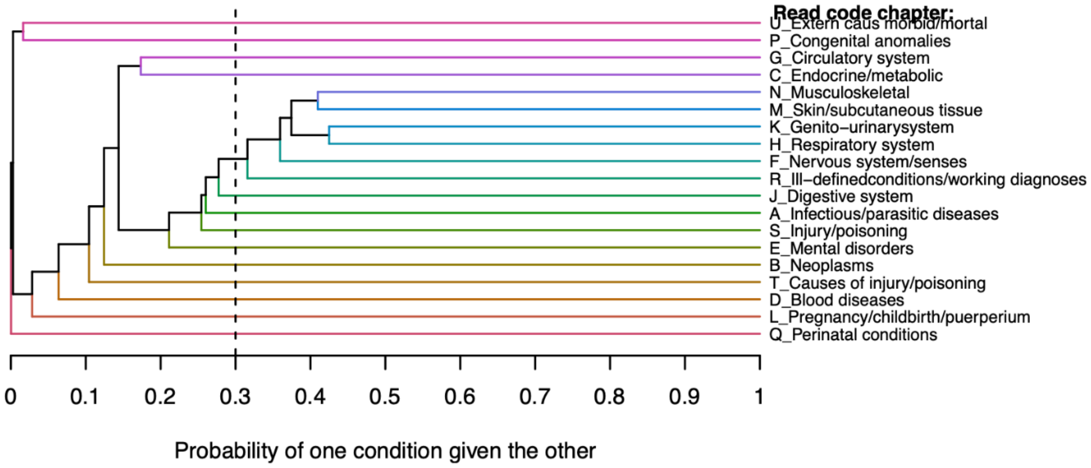

Age 50, women: matched controls, fu=10 years (complete linkage)

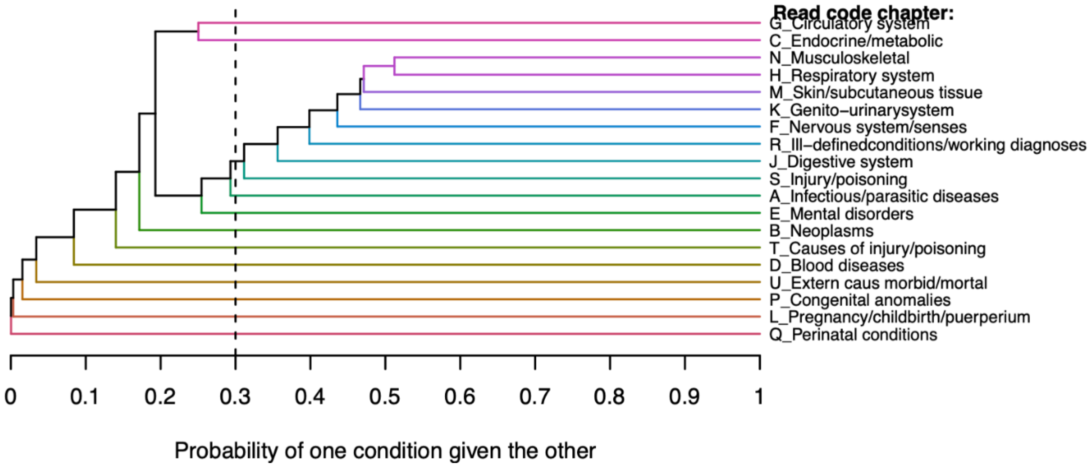

Supplementary Figure 14(H). Asthma cluster differences with 1, 5 and 10 years of [illegible]

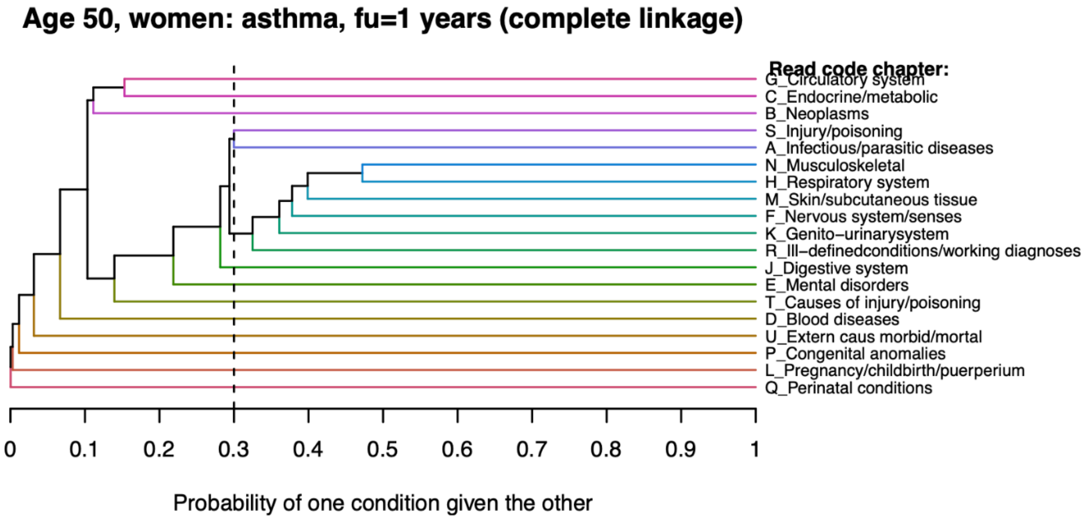

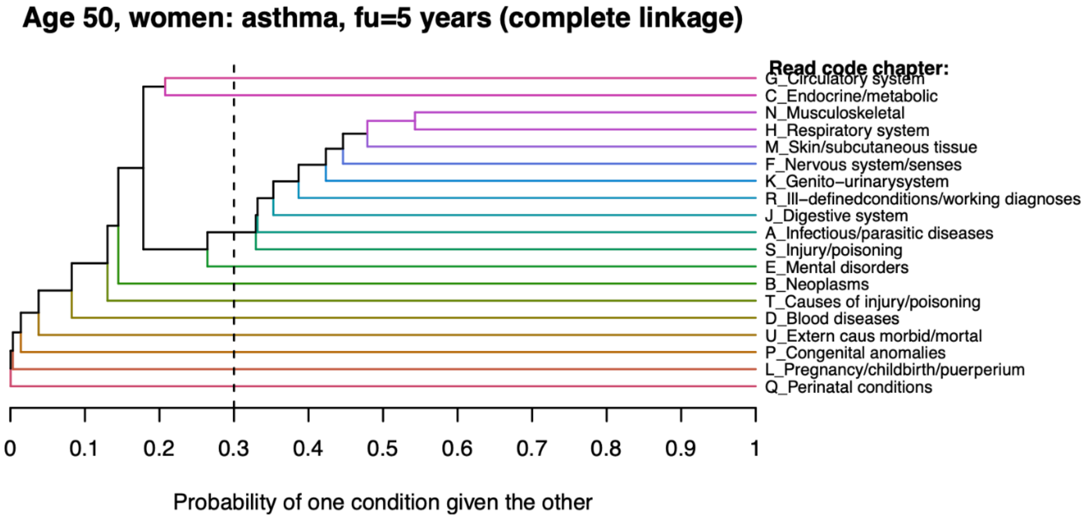

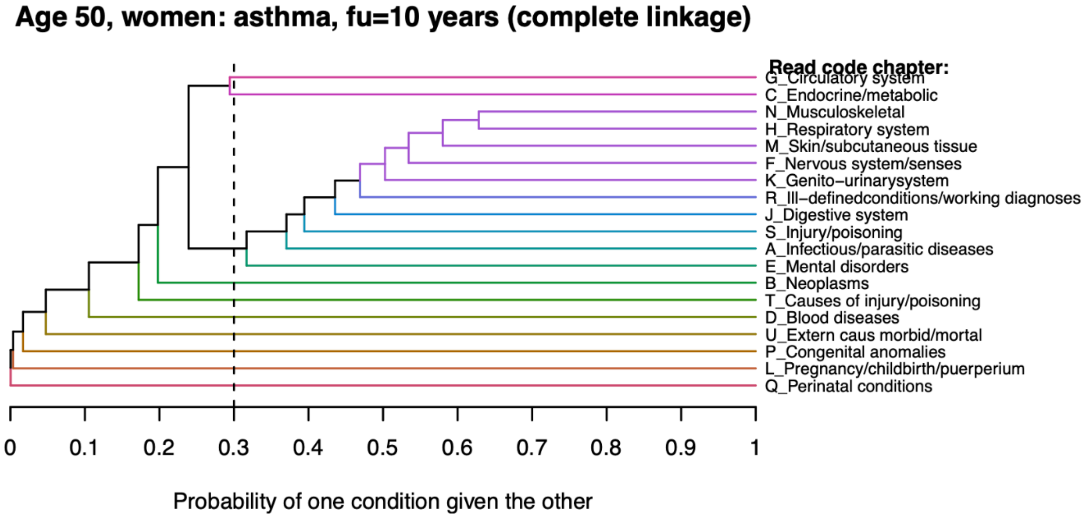

Supplementary Figure 15(A). Eczema cluster differences with 1, 5 and 10 years of followup

Age 18, men: matched controls, fu=1 years (complete linkage)

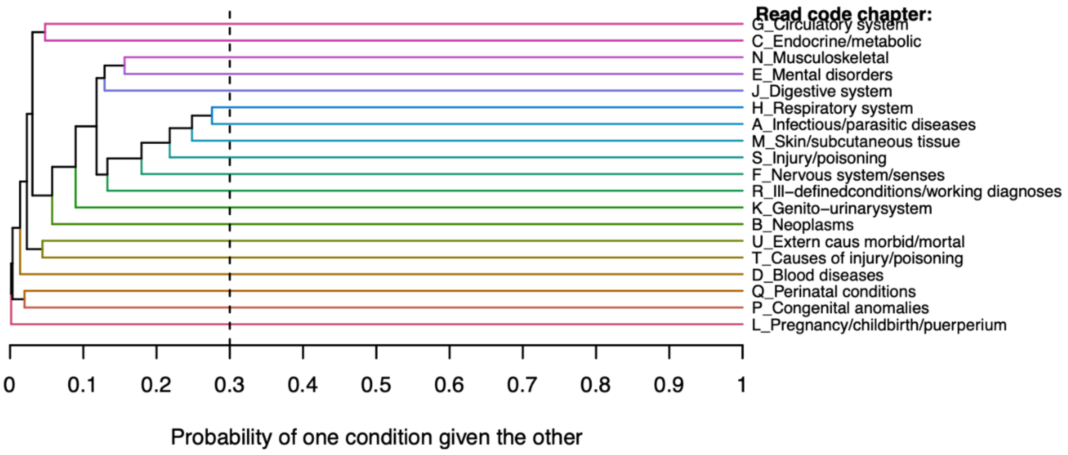

Age 18, men: matched controls, fu=5 years (complete linkage)

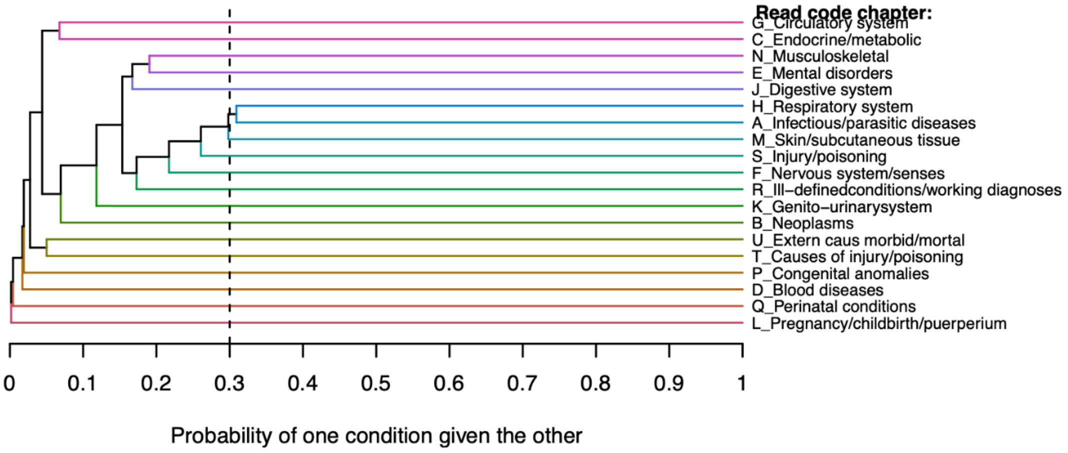

Age 18, men: matched controls, fu=10 years (complete linkage)

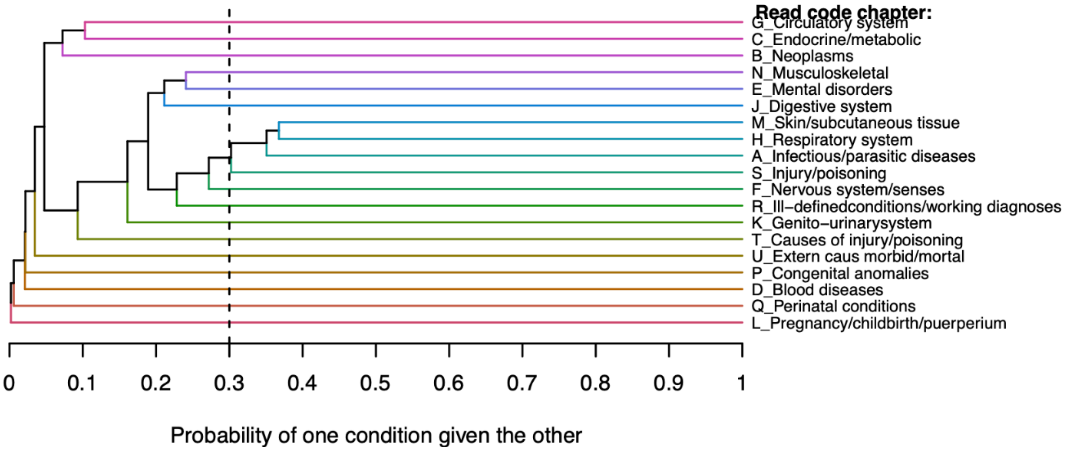

Supp. Figure 15(B). Eczema cluster differences with 1, 5 and 10 years of followup

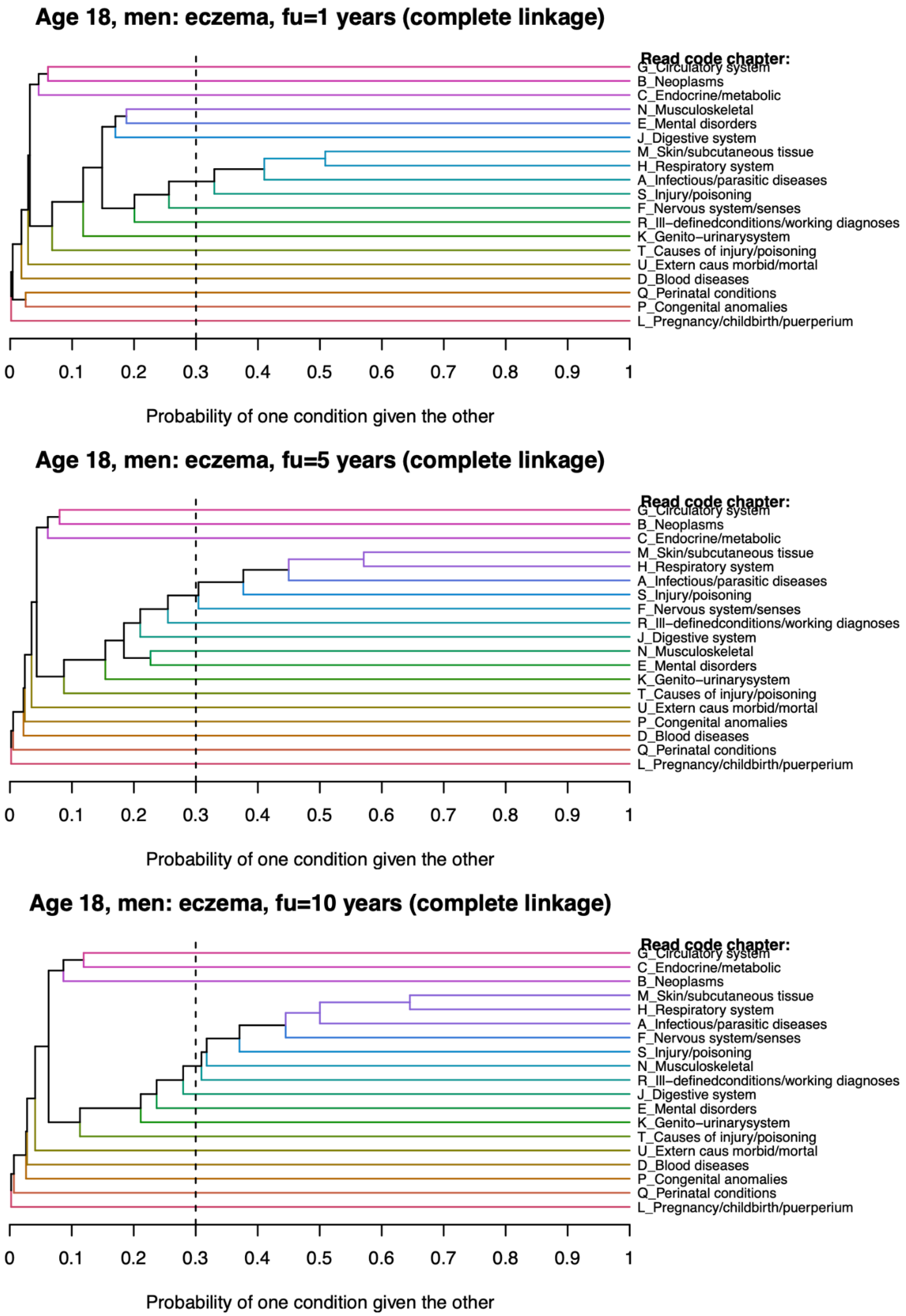

Supplementary Figure 15(C). Eczema cluster differences with 1, 5 and 10 years of followup

Age 50, men: matched controls, fu=1 years (complete linkage)

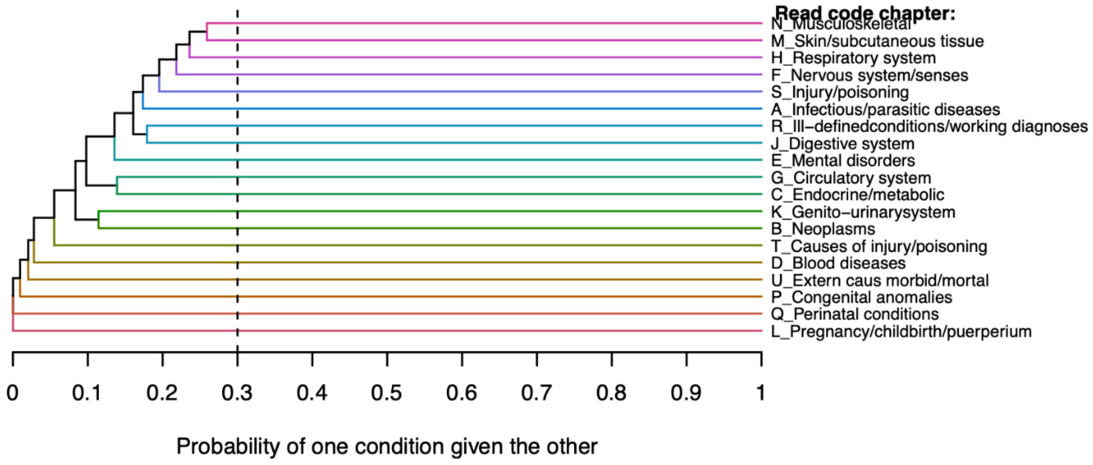

Age 50, men: matched controls, fu=5 years (complete linkage)

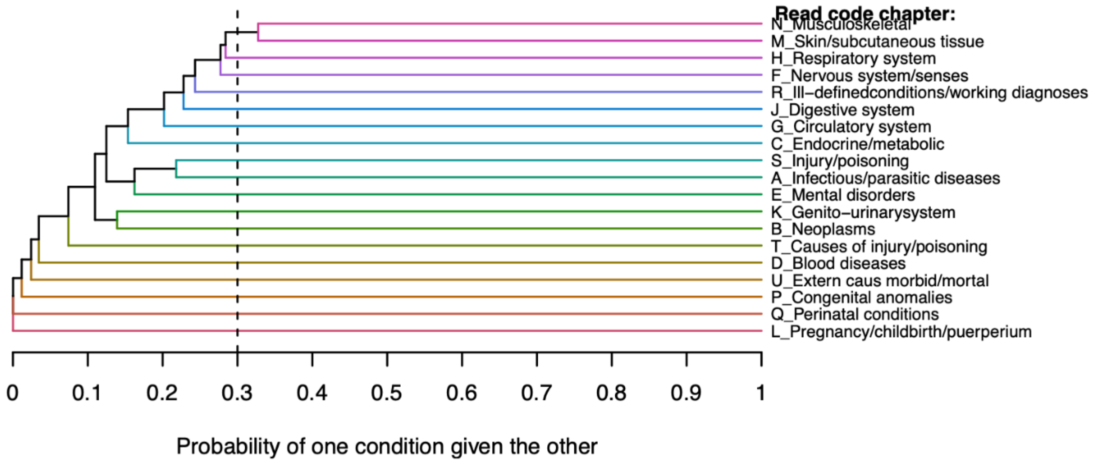

Age 50, men: matched controls, fu=10 years (complete linkage)

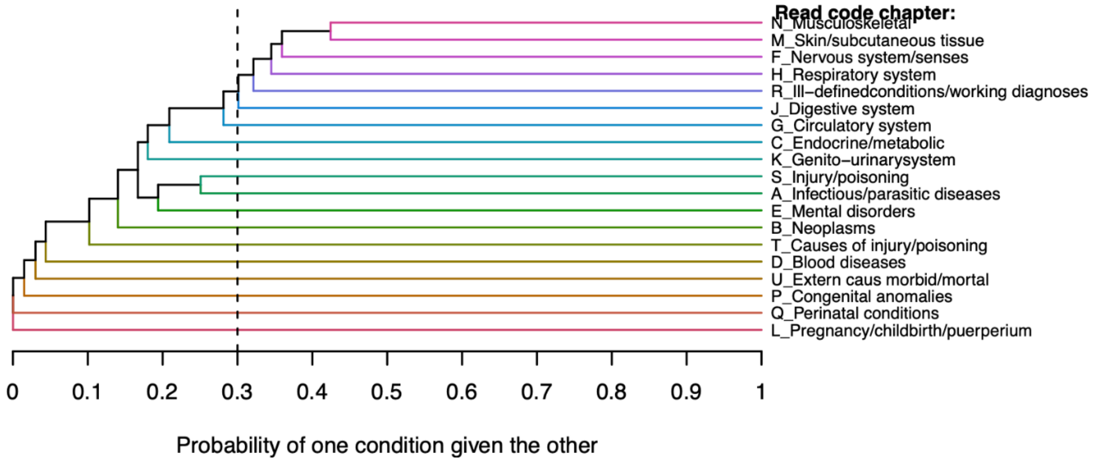

Supplementary Figure 15(D). Eczema cluster differences with 1, 5 and 10 years of followup

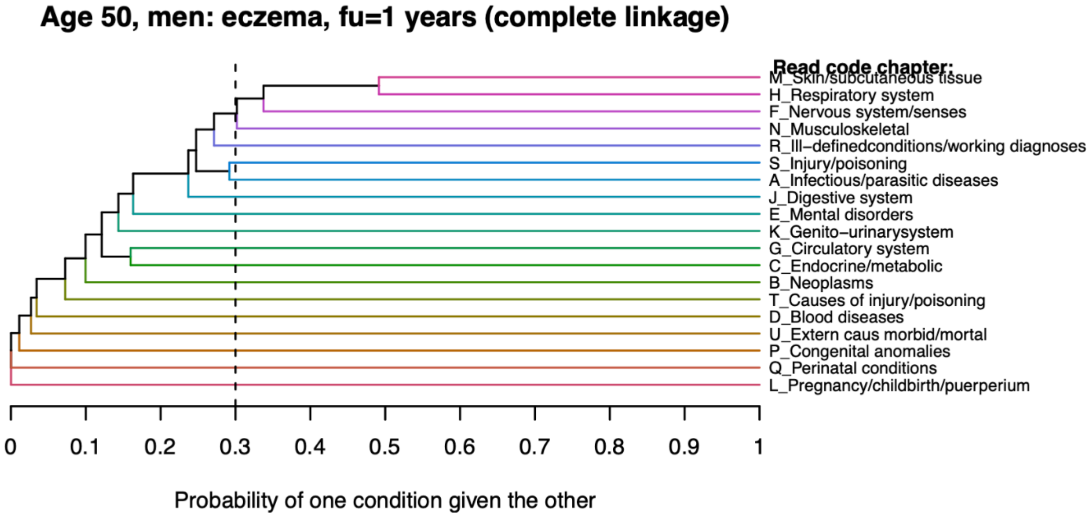

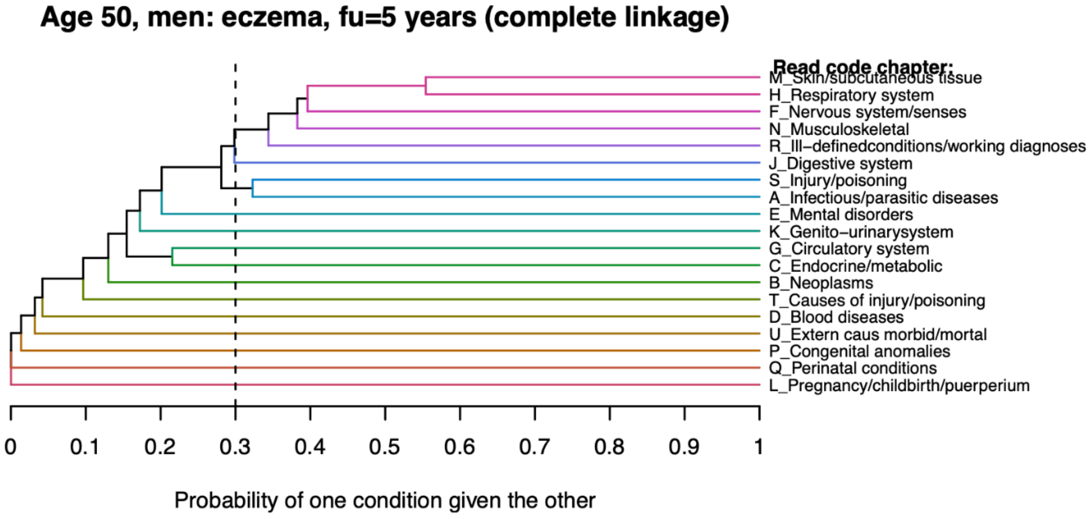

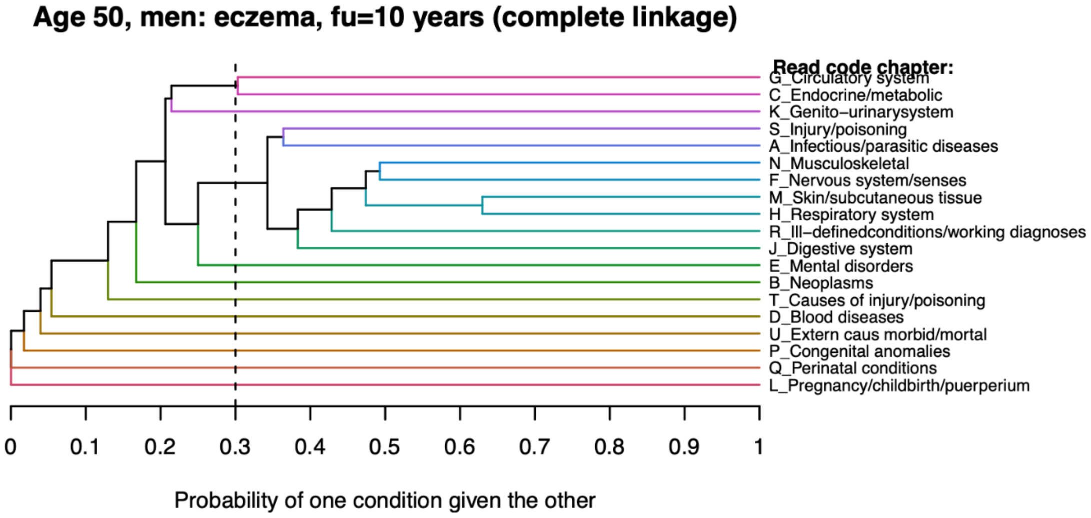

Supplementary Figure 15(E). Eczema cluster differences with 1, 5 and 10 years of

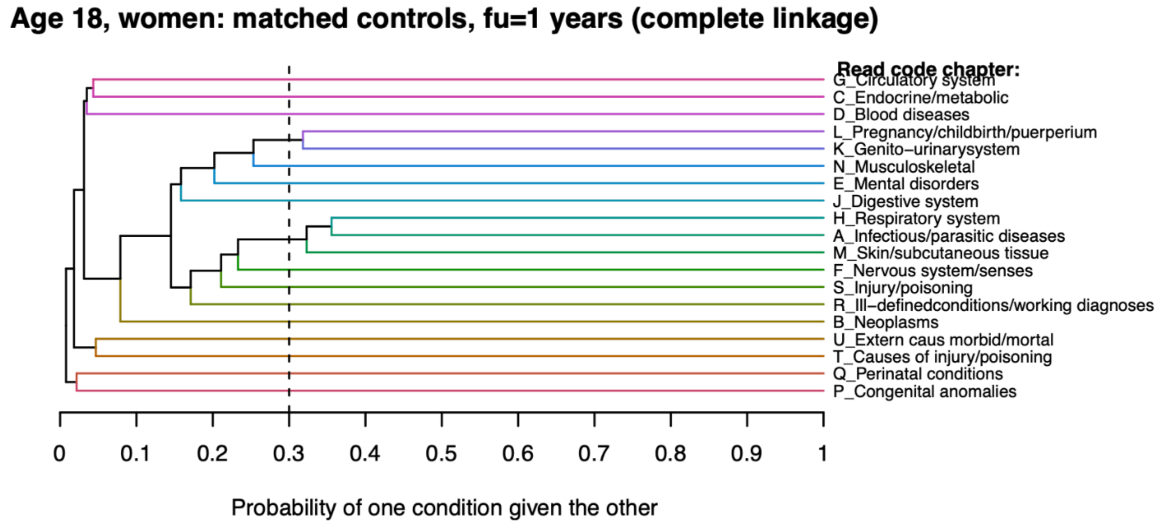

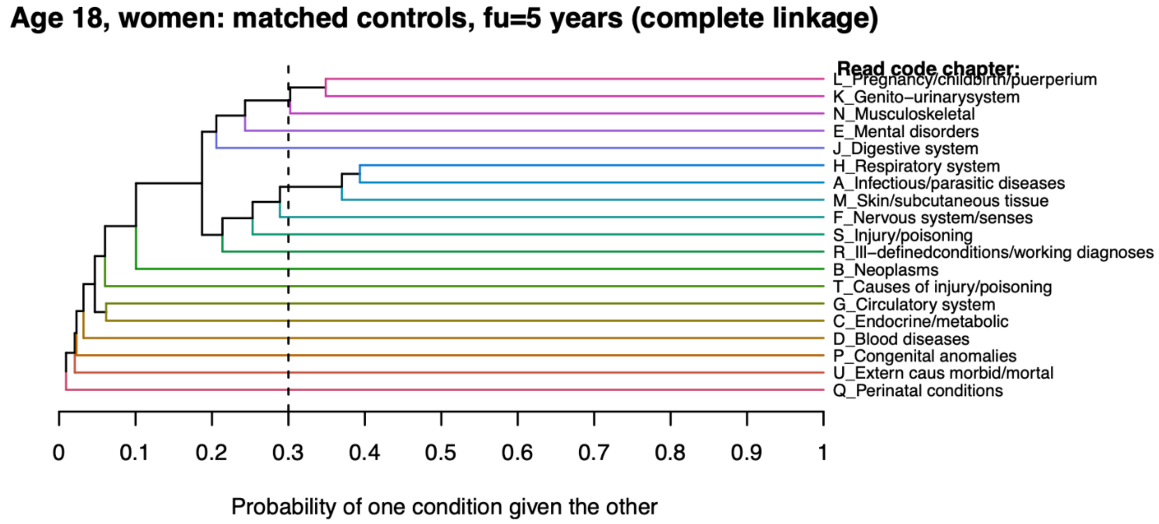

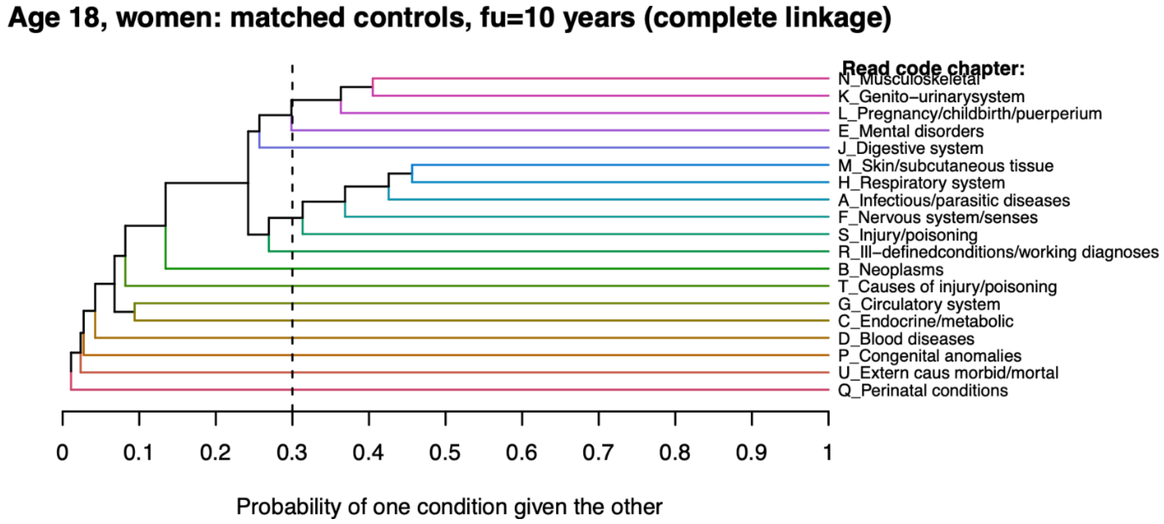

Supplementary Figure 15(F). Eczema cluster differences with 1, 5 and 10 years of followup

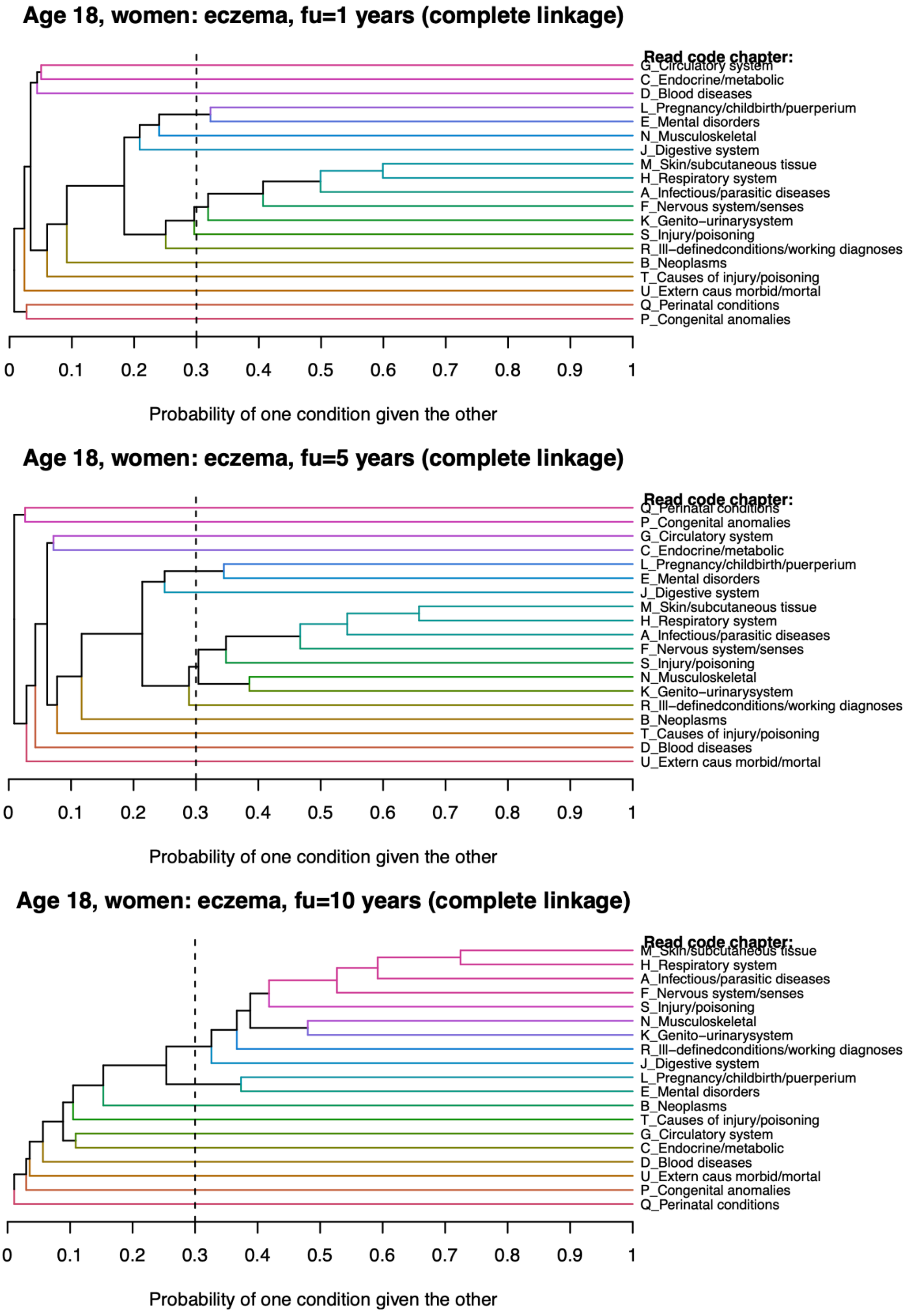

Supplementary Figure 15(G). Eczema cluster differences with 1, 5 and 10 years of followup

Age 50, women: matched controls, fu=1 years (complete linkage)

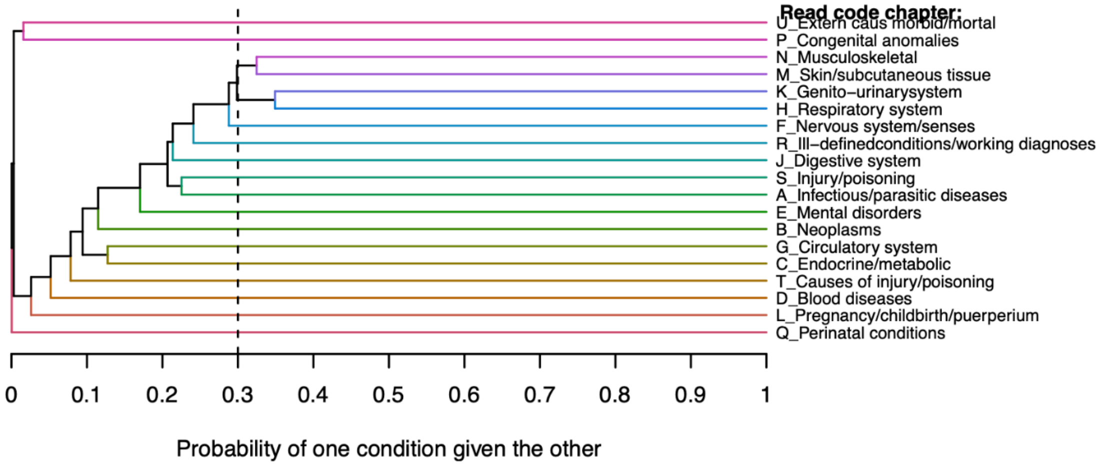

Age 50, women: matched controls, fu=5 years (complete linkage)

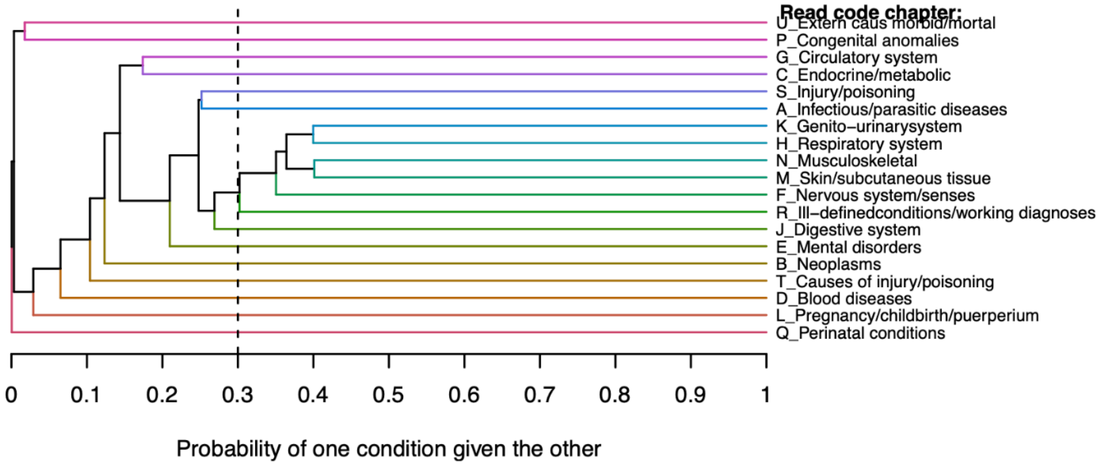

Age 50, women: matched controls, fu=10 years (complete linkage)

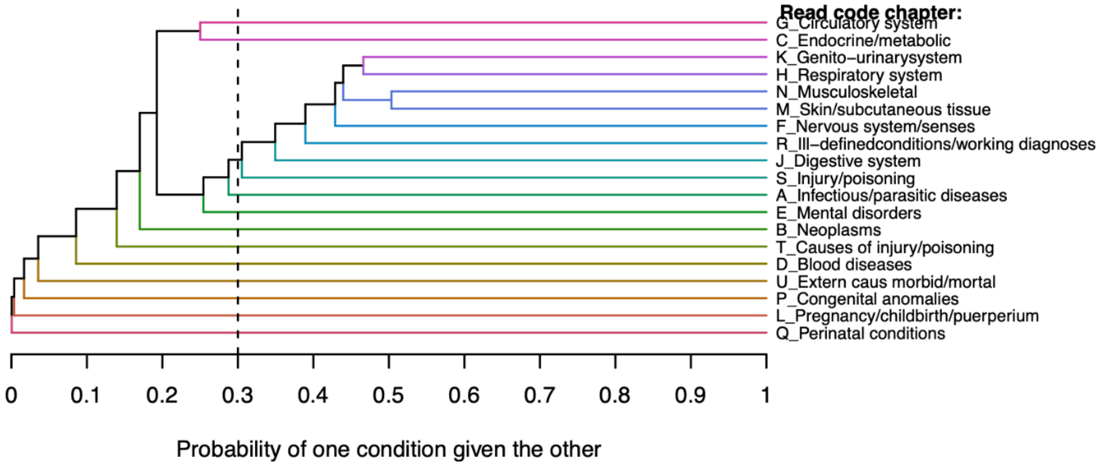

Supplementary Figure 15(H). Eczema cluster differences with 1, 5 and 10 years of followup

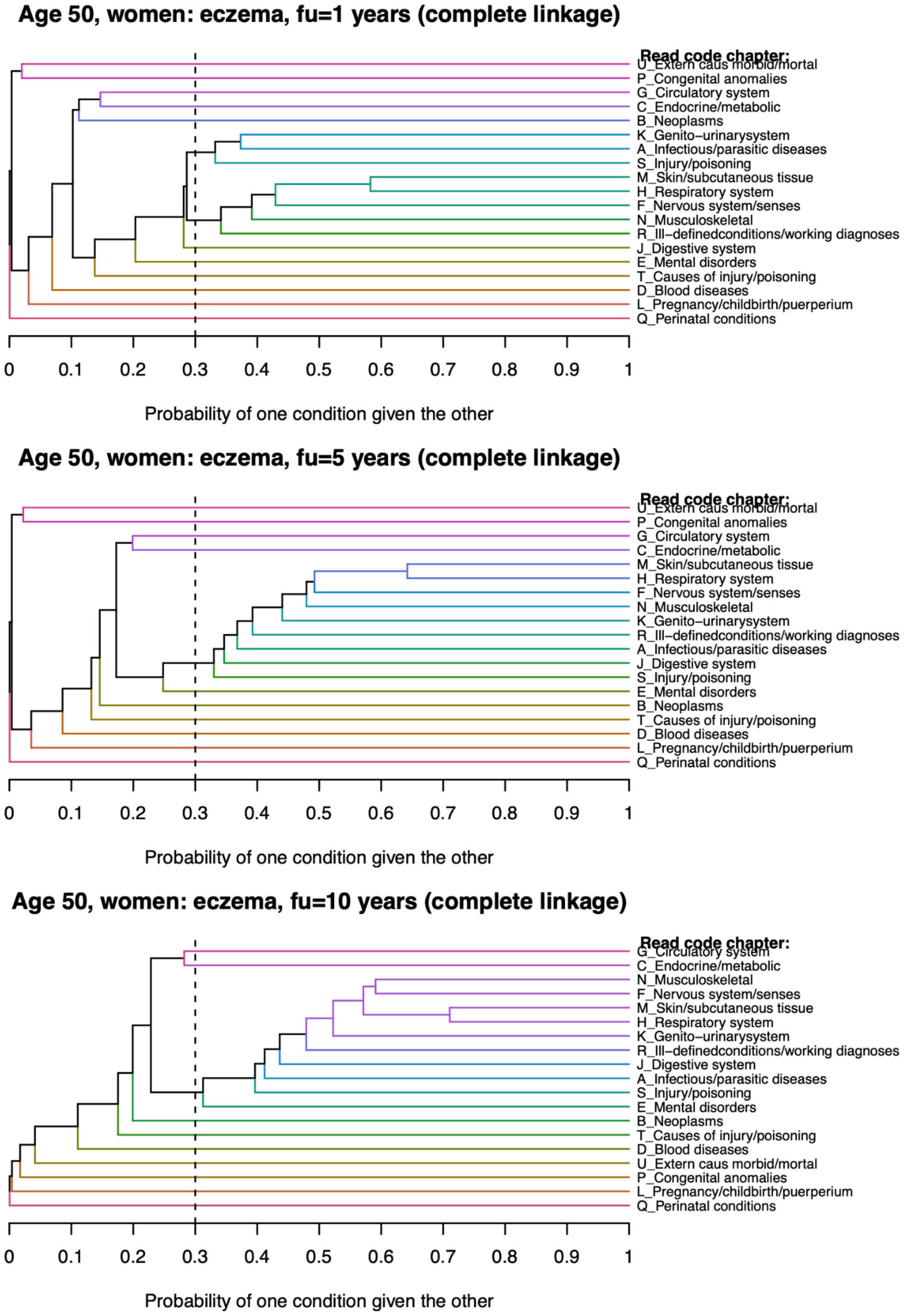

Supplementary Figure 16. Reproduction of Figure 2 using Ward linkage instead of complete linkage in the hierarchical clustering algorithm.

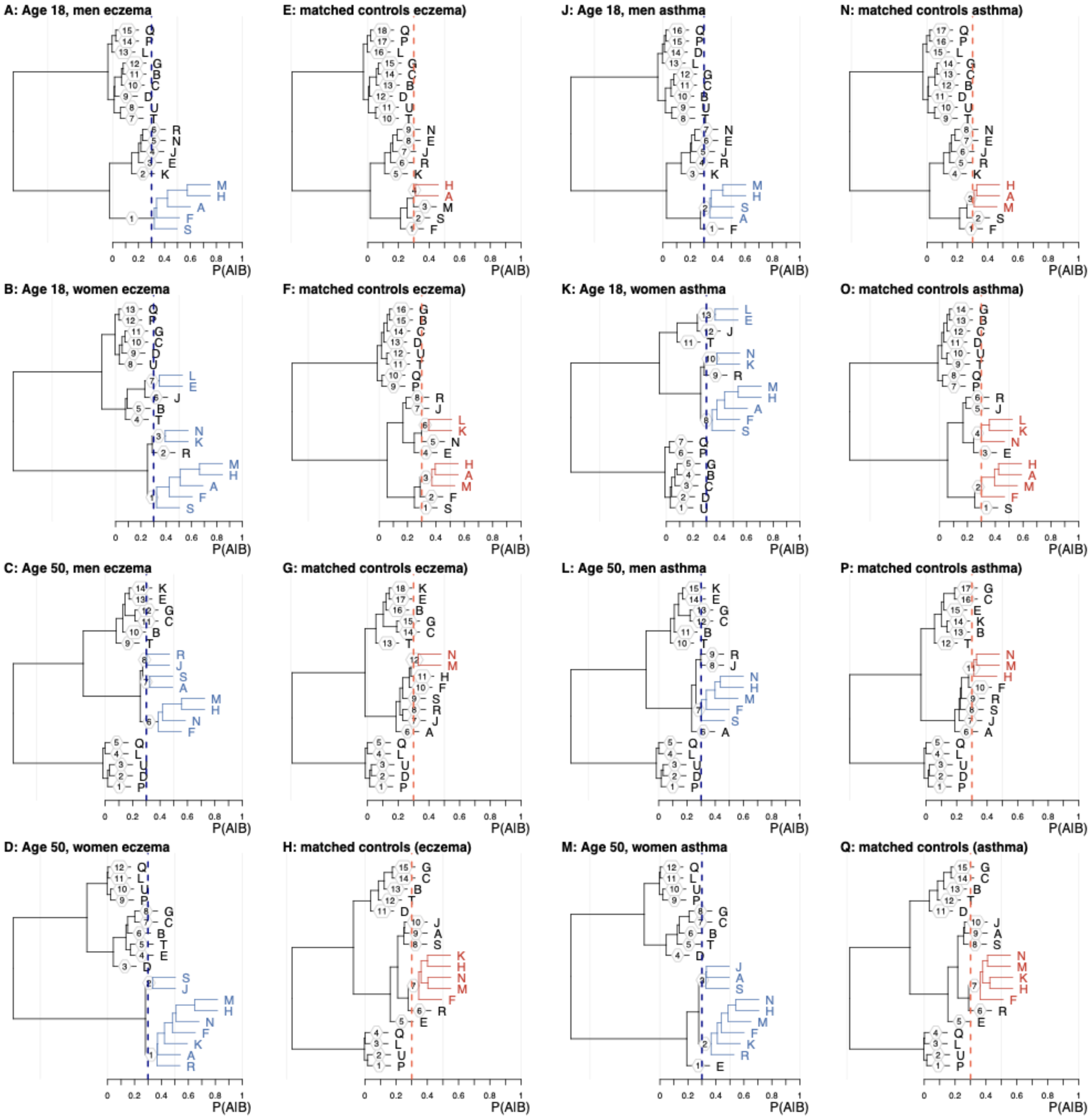

Supplementary Figure 17: graphical representation of Jaccard distance between two Read code chapters in the case of high prevalence of both chapters (examples 1 and 2), and when one chapter has low prevalence (examples 3 and 4)

The Jaccard distance measures the dissimilarity between two sets.

$$d_J(A,B) = 1 - J(A,B) = \frac{|(A \cup B)| - |(A \cap B)|}{|(A \cup B)|}$$

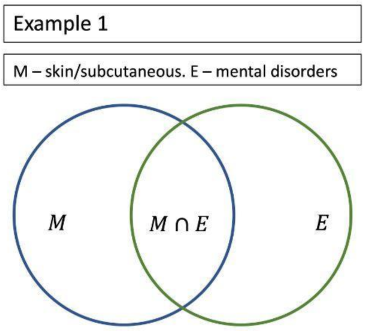

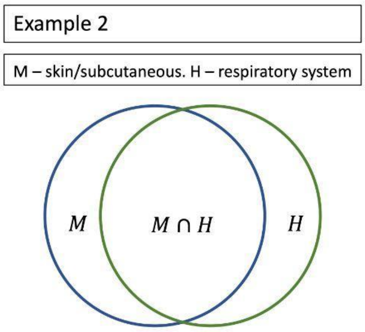

In these examples the larger area of $|M \cap H|$ compared to $|M \cap E|$ means that the Jaccard distance between chapters M and H is **smaller** than between M and H.

However, the calculation is sensitive to the size of the two sets. In these examples, there are relatively few records in chapters D and T.

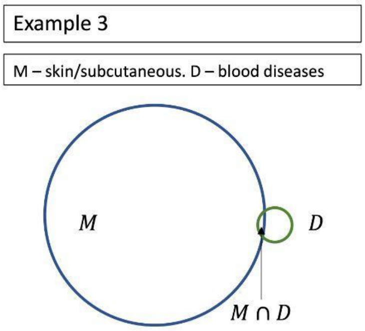

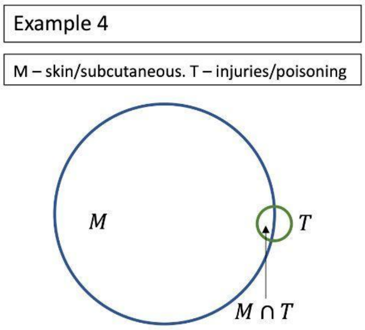

In these examples, even though the area $|M \cap T|$ is larger than $|M \cap D|$, the Jaccard distance would be similar because these areas of intersection are relatively small compared to areas of union, $|M \cup T|$ and $|M \cup D|$ respectively.
